# Inferring Dynamic Signatures of Microbes in Complex Host Ecosystems

**DOI:** 10.1371/journal.pcbi.1002624

**Published:** 2012-08-02

**Authors:** Georg K. Gerber, Andrew B. Onderdonk, Lynn Bry

**Affiliations:** 1 Department of Pathology, Brigham and Women's Hospital, Boston, Massachusetts, United States of America; 2 Department of Pathology, Harvard Medical School, Boston, Massachusetts, United States of America; University of California Davis, United States of America

## Abstract

The human gut microbiota comprise a complex and dynamic ecosystem that profoundly affects host development and physiology. Standard approaches for analyzing time-series data of the microbiota involve computation of measures of ecological community diversity at each time-point, or measures of dissimilarity between pairs of time-points. Although these approaches, which treat data as static snapshots of microbial communities, can identify shifts in overall community structure, they fail to capture the dynamic properties of individual members of the microbiota and their contributions to the underlying time-varying behavior of host ecosystems. To address the limitations of current methods, we present a computational framework that uses continuous-time dynamical models coupled with Bayesian dimensionality adaptation methods to identify time-dependent signatures of individual microbial taxa within a host as well as across multiple hosts. We apply our framework to a publicly available dataset of 16S rRNA gene sequences from stool samples collected over ten months from multiple human subjects, each of whom received repeated courses of oral antibiotics. Using new diversity measures enabled by our framework, we discover groups of both phylogenetically close and distant bacterial taxa that exhibit consensus responses to antibiotic exposure across multiple human subjects. These consensus responses reveal a timeline for equilibration of sub-communities of micro-organisms with distinct physiologies, yielding insights into the successive changes that occur in microbial populations in the human gut after antibiotic treatments. Additionally, our framework leverages microbial signatures shared among human subjects to automatically design optimal experiments to interrogate dynamic properties of the microbiota in new studies. Overall, our approach provides a powerful, general-purpose framework for understanding the dynamic behaviors of complex microbial ecosystems, which we believe will prove instrumental for future studies in this field.

## Author Summary

Microbes colonize the human body soon after birth and propagate to form rich ecosystems. These ecosystems play essential roles in health and disease. Recent advances in DNA sequencing technologies make possible comprehensive studies of the time-dependent behavior of microbes throughout the body. Sophisticated computer-based methods are essential for the analysis and interpretation of these complex datasets. We present a computational method that models how human microbial ecosystems respond over time to perturbations, such as when subjects in a study are treated with a drug. When applied to a large publicly available dataset, our method yields new insights into the diversity of dynamic responses to antibiotics among microbes in the human body. We find that within an individual, sub-populations of microbes that share certain physiological roles also share coordinated responses. Moreover, we find that these responses are similar across different people. We use this information to improve the experimental design of the previously conducted study, and to develop strategies for optimal design of future studies. Our work provides an integrated computer-based method for automatically discovering patterns of change over time in the microbiota, and for designing future experiments to identify changes that impact human health and disease.

## Introduction

The human gut harbors a dense and complex microbial ecosystem. Our ability to extensively characterize the microbiota has greatly increased in the last several years, due to lower costs and technical improvements in both DNA sequencing [1] and bioinformatics tools [2], [3]. High-throughput sequencing-based studies of the microbiota generally analyze regions of the conserved 16S ribosomal subunit gene [4], or use shotgun sequencing to sample the entire repertoire of genes present in a complex, mixed population of microbes [5]–[8]. These studies, of either human subjects or animal models, have uncovered intriguing associations between the composition of the gut microbiota and various diseases, including obesity [8], inflammatory bowel disease [7], [9], and multiple sclerosis [10].

Longitudinal studies of the microbiota are particularly important for further advancing the field [3], [5], [6], [11]–[13]. The majority of such longitudinal studies have been observational, monitoring the composition of the flora in healthy individuals over time at various body sites [3], [5], [6], [11]. Such observational studies are valuable for understanding natural variations in commensal communities, as well as capturing rarer events such as onset and resolution of acute disease in the host. Additionally, interventional studies have been performed, in which subjects were intentionally exposed to agents that perturb the microflora, with subsequent evaluation of changes in host ecosystems over time [12], [13]. Such interventional studies hold promise for discovering the mechanisms by which microbes interact with one another and the host, and to define how sub-communities of micro-organisms may cause or protect against disease.

To date, longitudinal studies of the microbiota have largely employed static analysis techniques that do not adequately capture the dynamic nature of the data. The most common types of analyses employed involve either computation of diversity measures, such as the Shannon-Weaver diversity index [14], at each data point, or measures such as Unifrac [15] or Bray-Curtis dissimilarity [16], which characterize pair-wise relationships between data points. These techniques have proven useful for uncovering certain trends in longitudinal data [3], [6], [8], [11], [12]. However, these techniques treat longitudinal data as a collection of static snapshots, and ignore inherent ordering and other temporal dependencies.

We developed a probabilistic model and inference algorithm, called Microbial Counts Trajectories Infinite Mixture Model Engine (MC-TIMME), which provides a unified framework for analyzing the dynamic behavior of the microbiota captured via high-throughput sequencing data. Our framework models time-varying counts of microbial taxausing exponential *relaxation processes*. Each relaxation process is characterized by a *transient effect level* (the amplitude of the process immediately after a perturbation), an *equilibrium level* (the amplitude of the process approached as time tends to infinity), and a *relaxation time* constant (the time required for the amplitude of the process to reach 33% of the transient effect level plus 67% of the equilibrium level, as measured on a logarithmic scale).Observed abundances of taxa are assumed to arise from an infinite mixture of *prototype signatures*. Each prototype signature is composed of a set of relaxation processes that model a response to multiple perturbations. Each reference operational taxonomic unit (refOTU) in the ecosystem(s) analyzed is probabilistically assigned to a prototype signature. Adaptive Bayesian techniques are used to model the dimensionality of prototype signatures and the extent of sharing of prototype signatures among refOTUs within and across ecosystem(s).The time-series of observed counts for a particular refOTU in an ecosystem is modeled through a generative process, in which the prototype signature to which the refOTU has been assigned is customized to an *individual signature* by addition of refOTU and time-point specific offset terms. Data is then generated from a discrete-valued noise model parameterized by the individual signature.

MC-TIMME enables several new types of analysis. The first type, termed Signature Diversity (SD) analysis, measures the variety of time-dependent microbial responses to perturbations to the host ecosystem(s). SD utilizes time-varying information, and is thus distinct from traditional static measures of ecological diversity [17], which characterize the complexity of a microbial community in terms of its constituent members at a single point in time. The second type, Relaxation Time Distribution (RTD) analysis, estimates the distribution of times required for members of the host ecosystem(s) to equilibrate after a perturbation event. This analysis summarizes the kinetics of responses, which is useful for understanding the phasing of changes within microbial ecosystems. The third type, Consensus Signature Group (CSG) analysis, identifies sub-communities of microbes within the larger ecosystem(s) that exhibit coordinated responses to a set of perturbations. This latter analysis provides information about which microbes may form functional sub-populations that affect the host or other microbial populations over time. Finally, MC-TIMME enables automated design of longitudinal studies of the microbiota. Our method couples information theoretic and Bayesian approaches to estimate from prior data the optimal sets of time-points to be sampled in future experiments. In this manner, data from a pilot study can be leveraged to develop optimized experimental designs for larger studies.

MC-TIMME has some similarities to previously published methods for analyzing other types of high-throughput data. Several studies have employed continuous-time models [18]–[21] or infinite mixture models [22], [23]to analyze time-series microarray data. Methods for optimal experimental design for time-series microarray data have also been described [24]. However, methods designed for analyzing gene expression data do not model dynamics inherent in complex microbial ecosystems, such as equilibrium reverting behavior. Further, these methods generally assume observed data are continuously valued and normally distributed, which is reasonable for microarray data, but not sequencing data, which consist of counts. Extensive statistical literature has documented that, for data consisting of discrete counts, direct modeling of the data yields superior results as compared to transforming data to continuous values (see e.g., [25]–[27]). This issue has been recognized for RNA Seq data, and several methods have been developed that use discrete-valued noise models [28]–[30]. Recently, Holmes *et al.* [31]presented a method for modeling microbial metagenomics counts data that also uses discrete-valued distributions. However, in contrast to MC-TIMME, these methods for analyzing RNA Seqor metagenomics count data use only static models.

To gain new understanding of dynamic changes in the human gut microbiota caused by antibiotic exposure, we applied MC-TIMME to data from a longitudinal study by Dethlefsen *et al.* [12]. Despite the profound effects antibacterial agents presumably have on commensal species *in vivo*, remarkably little is known about the rates at which these complex ecosystems recover or remain altered after cessation of a course of antibiotics. Further, it remains poorly understood how antibiotic-induced changes in the microbiota affect underlying host physiology, including enhanced susceptibilities to other pathogens [32], disease states such as allergic and auto-immune responses [33], [34], and acute or chronic effects on host diet and metabolism [35].To date, the Dethlefsen *et al.* study provides the longest time-series systematically monitoring the effects of antibiotics on human gut commensals. In this study, human subjects were given two spaced five day courses of oral ciprofloxacin, a broad-spectrum antibiotic that targets the DNA gyrase and topoisomerases of many microbial species [36]–[40]. Subjects' gut microbiota was monitored at 50+ time-points over nearly a year, by sequencing 16S rRNA gene signatures present in stool samples.

The remainder of the manuscript is organized as follows. First, we provide additional background on the Dethlefsen *et al.* dataset that we re-analyzed. Second, we describe the MC-TIMME framework, including our model of dynamics, inference algorithm, and automated experimental design method. Third, we apply MC-TIMME to the Dethlefsen *et al.* data to demonstrate the utility of Signature Diversity (SD), Relaxation Time Distribution (RTD), and Consensus Signature Group (CSG) analyses, and experimental design methods. Our results provide new quantitative insights into how antibiotic exposures affect the human gut microbiota, and how these dynamic alterations may influence the host.

## Methods

### Dataset Summary

Dethlefsen *et al.* [12] examined the microbiota from stool samples of three human subjects over a 10 month period, using Roche 454 high-throughput sequencing of PCR amplicons spanning the V1, V2 and V3 regions of 16S rRNA genes, producing a total of approximately 5 million reads. During the study, each subject received two separate 5 day courses of oral ciprofloxacin. Stool samples were collected daily during, one week prior, and one week after the antibiotics courses, but otherwise were collected less regularly throughout the study. Dethlefsen *et al.* combined the sequencing reads from all subjects to produce 2,582 reference operational taxonomic units (refOTUs). However, they found that only a few hundred refOTUs were present at consistently detectable levels in at least one sample per subject. In order to focus our analysis on refOTUs above the threshold of detection of the sequencing assay, we required that refOTUs have ≥5 counts for ≥10 time-points. This resulted in a total of 218 refOTUs for subject D, 261 for subject E, and 277 for subject F. Dethlefsen *et al.* taxonomically labeled refOTUs using the Silva 100 Small Subunit Reference database [41], and UClust [42] with a minimum best hit similarity of 95%. Data files containing counts and taxonomic labels for refOTUs were downloaded from the website linked to the original publication; DNA sequences for each refOTU were not available.

### MC-TIMME

#### Overview

**Figure 1:**
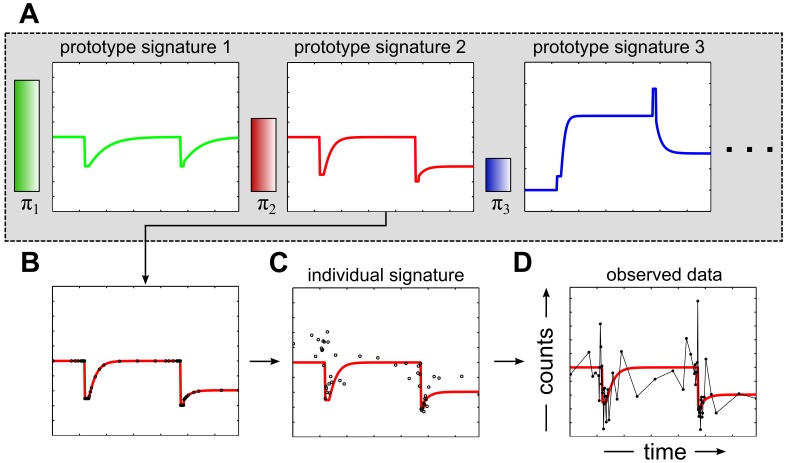
Schematic of the Microbial Counts Trajectories Infinite Mixture Model Engine (MC-TIMME) generative probabilistic model. Observed data of time-series of sequencing counts for reference operational taxonomic units (refOTUs) are assumed to arise from a multi-level generative probabilistic mixture model.(**A**)Infinite mixture over latent prototype signatures (green, red and blue solid lines),which specify models of dynamics continuous in both time and amplitude. The horizontal axis for each prototype signature represents time, and the vertical axis represents amplitude. Prototype signatures may adapt their dimensionality, which is shown increasing from left to right. The variables π_i_ and associated shaded bars represent prior probabilities for choosing among prototype signatures. (**B**)For each refOTU, a prototype signature is probabilistically chosen and sampled at discrete observed time-points. (**C**)Experiment and refOTU specific variables are added to the selected prototype signature to create an individual signature. (**D**) Observed data, consisting of sequencing counts, is generated through a discrete-valued noise model parameterized by individual signatures generated in step C.

MC-TIMME uses a Bayesian nonparametric hierarchical generative probability model as depicted in Figure 1. The ecosystem(s) analyzed are assumed to be decomposable into probabilistic mixtures of functions, continuous in both time and value, termed prototype signatures (Figure 1A). The data generation process probabilistically assigns each refOTU to a prototype signature, which induces a clustering of refOTUs into groups that share similar dynamics. The continuous-time prototype signature is then sampled at observed time-points (Figure 1B). The prototype signature is converted to an individual signature(Figure 1C) through a refOTU specific scaling term, and an experiment-wide normalization term that accounts for the total numbers of sequencing reads across experiments. Finally, the observed data for each refOTU, a time-series of counts, is generated from the individual signature through a discrete-valued noise model (Figure 1D).

**Figure 2:**
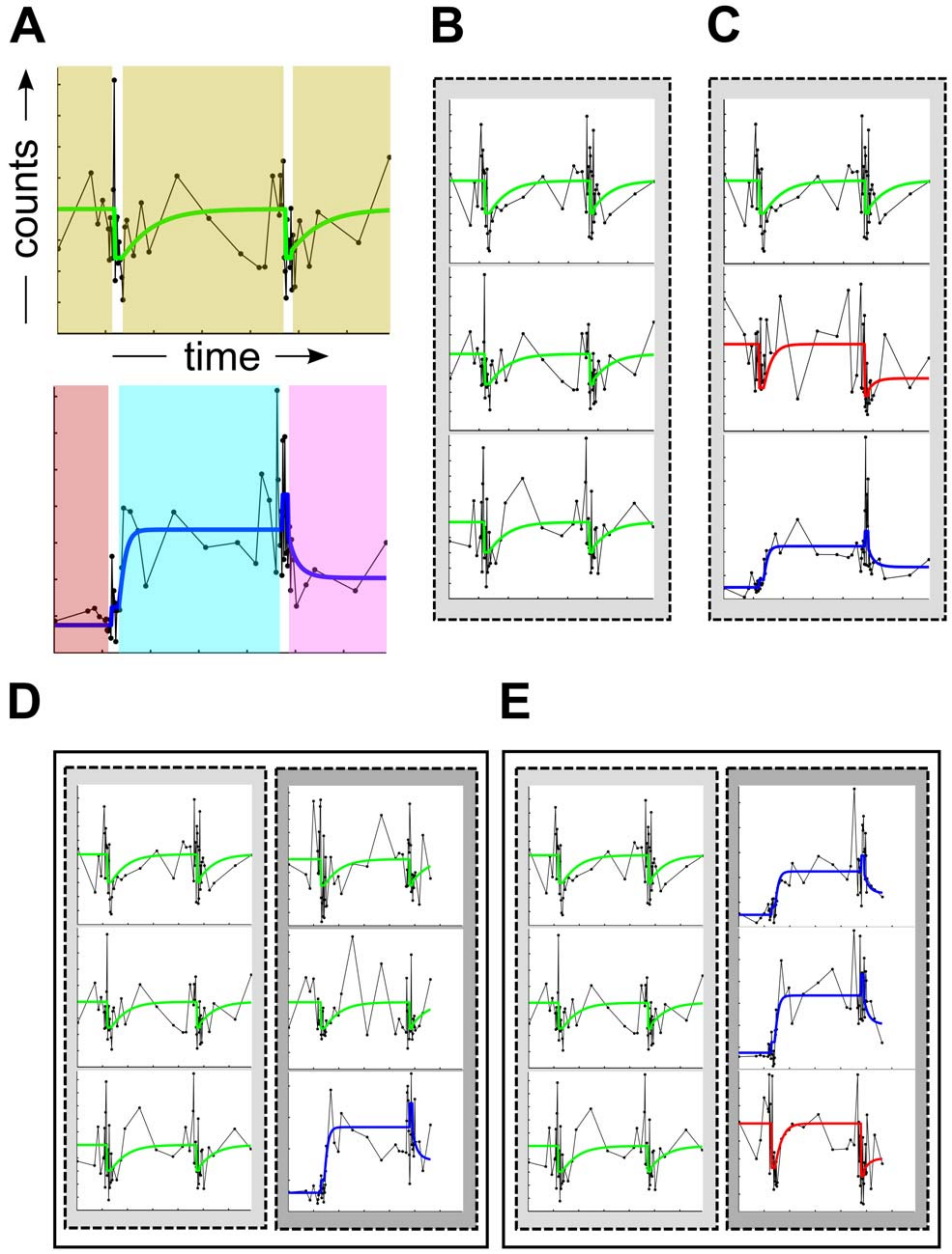
Schematics of microbial ecosystems illustrating Signature Diversity scores at multiple levels of resolution. The panels depict examples of simplified microbial ecosystems measured over time, to illustrate three levels of Signature Diversity (SD) scores computed by the Microbial Counts Trajectories Infinite Mixture Model Engine (MC-TIMME) framework. (**A**) Intra-signature diversity (SD1), characterizes the dimensionality of each prototype signature. The top panel depicts a prototype signature with a lower SD1 score than the prototype signature in the bottom panel, which exhibits different equilibrium levels and relaxation time constants on each of the shaded intervals. (**B–C**) Intra-ecosystem signature diversity (SD2), characterizes the extent of prototype signature sharing among taxa within a host ecosystem. Host ecosystem (B) has a lower SD2 score than (C), because all taxa in (B) share the same prototype signature. (**D–E**) Inter-ecosystem signature diversity (SD3), characterizes the extent of prototype signatures haring across ecosystems. Each panel depicts two ecosystems. The two ecosystems in (D) have a lower SD3 score than those in (E), because more prototype signatures are shared between the ecosystems in D.

MC-TIMME adaptively learns three levels of Signature Diversity (SD) as depicted in Figure 2. These three levels are: (SD1) intra-signature (Figure 2A), or the dimensionality of each prototype signature, (SD2) intra-ecosystem (Figures 2B,2C), or the diversity of prototype signatures among the taxa within an ecosystem, and (SD3) inter-ecosystem (Figures 2D,2E), or the diversity of prototype signatures across multiple host ecosystems. For SD1 adaptive learning, MC-TIMME employs latent variables that control the number of parameters that specify each prototype signature. For SD2, MC-TIMME incorporates Dirichlet Process infinite mixture models, which effectively adjust the number of prototype signatures used to model the system. ForSD3, MC-TIMME maps experiments from different ecosystems to the same time-scale for simultaneous analysis, facilitated by the continuous-time model of dynamics.

The MC-TIMME model is fully Bayesian, and we thus seek to infer the posterior probability distribution of the model variables given the data. However, the posterior distribution is not computable in closed form, making exact inference intractable. Instead, we approximate the posterior distribution using Markov Chain Monte Carlo (MCMC) methods, and then compute various summary statistics. Below, we provide further information on the MC-TIMME model and associated algorithms; see Protocol S1 for complete details.

Sample data, instructions, and GPLv3 licensed Matlab™ (MathWorks, Natick, MA) source code for MC-TIMME are available at https://sourceforge.net/projects/mctimme/. The implementation provided will process datasets with an arbitrary number of experimental subjects or perturbations, as specified through user supplied data files.

#### Dynamical model for antibiotic pulses

Prior longitudinal studies have qualitatively described several key dynamic properties of the gut microbiota [3], [12]. First, abundances of individual microbes within a stable ecosystem are constrained around average levels, despite day-to-day temporal variability. Second, when the ecosystem is perturbed, individual members exhibit varied responses. These responses are characterized by transient components that eventually decay toward an equilibrium state. Of note, this equilibrium state may differ from the pre-perturbation state. Third, the responses of micro-organisms within the ecosystem are dependent on one another. These dependencies may be multifactorial, including factors such as competition for nutrients and other essential resources, as well as common reactions to phases of the host immune response.

To model these phenomena for the Dethlefsen *et al.* data, we assume that the dynamics for each refOTU are characterized by piece-wise defined functions over five intervals delimited by the antibiotic treatments in the experiment (Figure S1). These five intervals are: (a) pre-antibiotic, (b) antibiotic treatment one, (c) post-antibiotic treatment one, (d) antibiotic treatment two, and (e) post-antibiotic treatment two.

Observed sequencing counts *y_sot_* for subject *s*, refOTU*o*, and at time *t* are assumed to be samples from the negative binomial distribution (NBD), a two parameter distribution [25]. The density function of the NBD can be parameterized in terms of its mean *m* and inverse shape parameter *ε*. Thus, for *y_sot_* the NBD density function is given by:


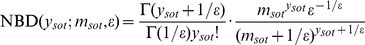


Let each prototype signature *k* be associated with a deterministic function f(*t,θ_k_*). We then specify the mean of the NBD *m_sot_* at time *t* for refOTU*o* in subject *s* assigned to prototype signature *k* as:


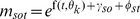


The variable *γ_so_* is a subject and refOTU specific offset that scales the baseline number of counts for refOTU*o* in subject *s*. The variable *φ_st_* is a subject and time-point specific offset that accounts for differences in the total number of sequencing reads among experiments for subject *s*.

For intervals (a), (b) and (d), we assume f(*t,θ_k_*) is constant valued, i.e., f*_a_*(•) = *μ_ka_*, f*_b_*(•) = *X_kb_*, and f*_d_*(•) = *X_kd_*. We assume that dynamics in interval (c), the period after the first antibiotic treatment and before the second, are specified by an exponential relaxation process:


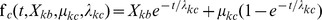


This process has initial value *X_kb_*, modeling the transient effect of the antibiotic treatment, and approaches equilibrium level *μ_kc_* with relaxation time *λ_kc_*.

For interval (e), the period after the second antibiotic treatment, we also assume an exponential relaxation process:


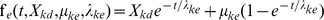


For the NBD inverse shape parameter, we assume it is equal to the same value, ε_1_, on intervals (a), (c) and (e), and equal to a different value, ε_2_, on the antibiotic treatment intervals (b) and (d).

To capture behavior of the microbiota that spans multiple temporal intervals, we model dependencies between interval parameters using random walks. For instance, the equilibrium level on interval (c), *μ_kc_*, for the prototype signature *k* is given by:


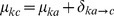


Here, *μ_ka_* is the pre-treatment equilibrium level, and *δ_ka→c_* is a random, normally distributed increment. We model relationships between the other interval parameters similarly, as described fully in Protocol S1.

To adapt the complexity of the dynamical models for prototype signatures, we introduce dimensionality changing variables, *c_kμ_* and *c_kλ_*. These variables are discrete-valued, and act as “switches” to control the number of equilibrium levels or relaxation time parameters utilized by each prototype signature.

#### Model inference and posterior distribution summarization

We approximate the posterior distribution of the MC-TIMME model using Markov Chain Monte Carlo (MCMC) methods. The DirichletProcess aspects of the model are handled with Gibbs sampling steps [43]. Updating prototype signature variables is more involved, because of temporally induced dependencies among variables, non-conjugacy of prior distributions, and the dimensionality changing variables. For these updates, we use combined Reversible Jump/Metropolis-Hastings steps. See Protocol S1 for complete details. We ran our MCMC algorithm with a burn-in of 10,000 iterations, and then collected every 10th sample for an additional 5,000 iterations. Convergence was evaluated using standard techniques as described in [44]. Eight independent MCMC runs were pooled for our final analysis. This produced a set of J = 4,000 posterior samples, which were then used to compute summary measures for Signature Diversity (SD), Relaxation Time Distribution (RTD), and Consensus Signature Group (CSG) analysis.

We define the Signature Diversity type 1 equilibrium level score, SD1_μ_, as the expected fraction of refOTUs with greater than one equilibrium level. We similarly define the SD1 relaxation time score, SD1_λ_, as the expected fraction of refOTUs with greater than one relaxation time constant. The SD1 scores are given by:


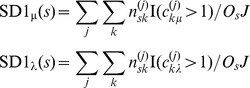


Here, I(•) denotes the indicator function, *n_sk_^(j)^* the number of refOTUs from subject *s* assigned to prototype signature *k* in MCMC sample *j*, and *O_s_* the number of refOTUs for subject *s*.

We define the Signature Diversity type 2 (SD2) score as the expected equivalent number of prototype signatures per 100 refOTUs. Because assignment of refOTUs to prototype signatures may be non-uniform, we use a measure that standardizes for this effect. The SD2 score is given by:


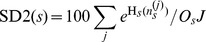


Here, H*_s_*(*n_s_^(j)^*) represents the entropy with respect to the distribution over assignments to prototype signatures for sample *j*, restricted to subject *s*. The exponentiated entropy yields the equivalent number of prototype signatures: the hypothetical number of prototype signatures, assuming a uniform assignment of refOTUs, which will yield an entropy equal to H*_s_*(*n_s_^(j)^*).

We define the Signature Diversity type 3 (SD3) score as a ratio of SD2 scores: SD2^D^, which is an SD2 score computed on a hypothetical combined ecosystem, and SD2^I^, which is the weighted average of independent SD2 scores computed on each ecosystem separately. These scores are given by:


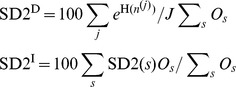


Here, H(*n^(j)^*) represents the entropy with respect to the distribution over assignments to prototype signatures for sample *j* for all subjects' ecosystems combined.

To characterize Relaxation TimeDistributions, we estimated probability density functions for relaxation time constants of all refOTUs, using the ksdensitykernel density estimation function in MatlabR2011b with the default options.

To characterize Consensus Signature Groups (CSGs), we used an agglomerative clustering method as described in [23]. For each pair of refOTUs*o* and *o′* in subjects *s* and *s′* (possibly the same subject) we computed:


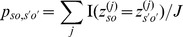


Here, *z^(j)^* is a random variable that specifies the assignment of a refOTU to a prototype signature for MCMC iteration *j*. The agglomerative clustering method successively merges CSGs based on average linkage using *p_so,s′o′_* as the similarity measure. Merging is stopped when the number of clusters reaches the expected number of prototype signatures, as calculated from all the MCMC samples. Consensus signatures and relaxation time constants for a CSG are then computed from the MCMC samples for all refOTUs belonging to that CSG.

To test for enrichment of Consensus Signature Groups for particular taxonomic labels, we used the following procedure. For each CSG, we computed *p*-values for the observed configuration of taxonomic labels of refOTUs at the order, family and genus levels, under the null hypothesis that configurations followed the hypergeometric distribution. We computed the false discovery rate (FDR) using the method of [45], and considered cases with FDR<0.05 significant.

### Experimental Design

Our approach is based on a Bayesian information theoretic formulation of the experimental design problem (see e.g., [46]–[48]). To define notation, suppose we are given a joint probability distribution p(Θ,*A(T)*) over model parameters Θ and possible data *A(T)* collected at a set of time-points *T*. Suppose we then perform experiments, which allow us to collect a dataset denoted *a*. This results in a gain in Shannon information that is given by:


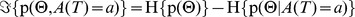


Here, H{*•*} denotes the differential entropy. Thus, we see that the gain in information is due to the difference in entropy between prior and posterior distributions.

The objective of our automated experimental design algorithm is then to choose the sampling times *T* that maximize the expected information gain over all possible data that could be observed at those time-points:


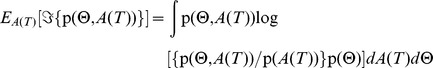


This is a high dimensional integral that is in general intractable. However, for a linear model with Gaussian noise, the integral can be written as [46], [49], [50]:


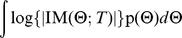


Here, IM denotes the Fisher information matrix, and |•| the determinant of the matrix. The integral can be approximated with a function g(*•*) of samples Θ^(*j*)^ from the priorp(Θ), yielding:


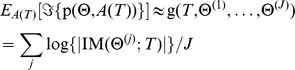


In the case of a Generalized Linear Model, a linear approximation can be calculated to yield a local Bayesian *D*-optimality measure [50]. We use this measure, as each prototype signature in MC-TIMME is a Generalized Linear Model if we condition on the appropriate parameters (see Protocol S1).

We estimate samples from p(Θ), the prior probability distribution over model parameters for future experiments, using a model learned from previously observed data. Specifically, we use 500 MCMC samples obtained from the posterior distribution of the MC-TIMME model conditioned on a set of observed data. We then use a greedy optimization algorithm with the Bayesian *D*-optimality function g(•)defined above, to generate experimental designs. See Protocol S1 for complete details.

## Results

### Algorithm Performance

MC-TIMME analyzed the complete Dethlefsen et al. dataset, consisting of 3 subjects with 50+ time-points each, in approximately 12 hours on an Intel Xeon E5507 2.27 GHz core. Figure S2 provides examples of individual signatures for refOTUs inferred by MC-TIMME. The subsequent sections detail our biological findings based on these analyses.

We also ran additional analyses to evaluate the sensitivity of our results to key model assumptions or features of the data. First, we tested the robustness of the model's dimensionality adaptation capability, which is a critical component of Signature Diversity scores. These tests showed no significant differences in our results when relevant model parameters were varied. Second, we tested the robustness of our results to noise. Because an equivalent gold standard experimental dataset does not exist, we generated simulated data for use in testing. For these simulations, we used all prototype signatures estimated by MC-TIMME from the full Dethlefsen et al. dataset as our gold standard, and then generated test datasets with varying amounts of added noise. When the amount of noise equaled that in the original dataset (coefficient of variation of ≈60% for counts), MC-TIMME recovered Signature Diversity scores with <≈10% error, and relaxation time constant estimates with ≈25% error for the first post-antibiotic exposure interval, and ≈40% error for the second interval; measures of consistency of assignment of refOTUs to prototype signatures showed ≈20% reduction in quality. Third and finally, we tested the sensitivity of our results to exclusion of each experimental subject. These tests showed error rates comparable to those from our simulations when noise levels were equal to those in the original dataset. See Protocol S1 for complete details. Overall, our model performance tests demonstrate that our results are robust to changes in relevant parameter settings, noise, and exclusion of experimental subjects.

### Signature Diversity

To characterize the diversity of responses of the microbiota to repeated antibiotic treatments, we calculated three types of Signature Diversity (SD) scores. As shown in Figure 2, each SD score (SD1 to SD3) measures the dynamic behavior of micro-organisms in the host ecosystem(s) at a different level of resolution. These scores take into account the responses over time for refOTUs, and thus provide new information about dynamic properties of the ecosystems studied, as compared to traditional static measures of ecological diversity [17].

**Figure 3:**
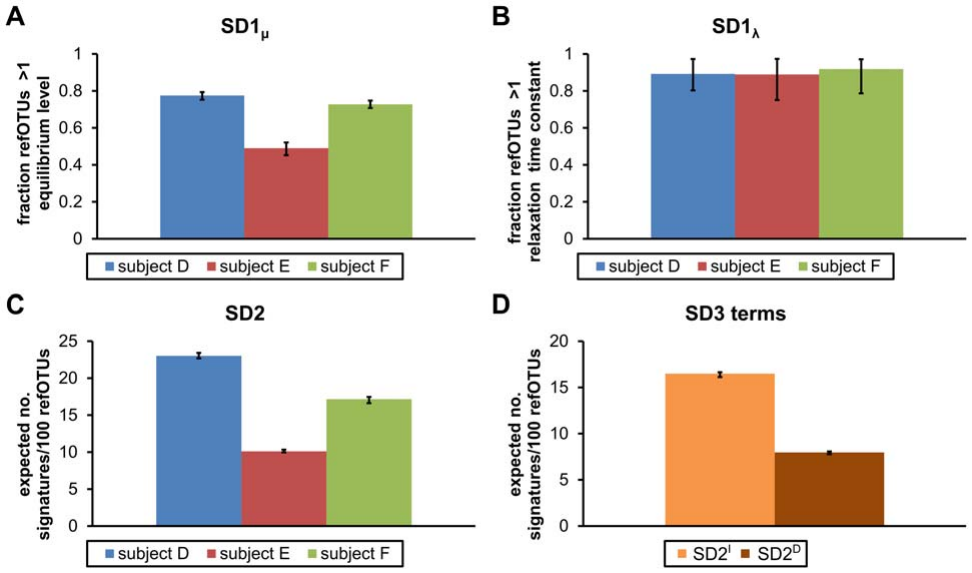
SignatureDiversity scores for gut microbiota of three human subjects treated twice with antibiotics. (**A**) Intra-signature diversity scores for equilibrium levels (SD1_μ_), which measure the expected fraction of reference operational taxonomic units (refOTUs) that change equilibrium levels in response to one or more of the antibiotic treatments. (**B**) Intra-signature diversity scores for relaxation times (SD1_λ_), which measure the expected fraction of refOTUs that exhibit different relaxation time constants after the antibiotic treatments. (**C**) Intra-ecosystem signature diversity scores (SD2), which measure the expected equivalent number of prototype signatures per 100 refOTUs. (**D**) The inter-ecosystem signature diversity score (SD3), which measures the degree of sharing of prototype signatures across host ecosystems, is a ratio of the SD2^D^to the SD2^I^ score. The SD2^D^score is computed on a hypothetical combined ecosystem, in which refOTUs from different subjects probabilistically share prototype signatures. The SD2^I^ score is a weighted average of SD2 scores computed on each subject separately.

The intra-signature diversity (SD1) scores for all three subjects in the Dethlefsen *et al.* study were >≈50% (Figures 3A, 3B), indicating that the majority of micro-organisms in these host ecosystems exhibited changes in equilibrium levels or relaxation times after one or both antibiotic treatments. As shown in Figures 3A and 3B, the SD1 score has two components: (1) SD1_μ_, which measures the expected fraction of refOTUs with changes in equilibrium levels between pre-treatment and at least one post-antibiotic treatment interval, and (2) SD1_λ_, which measures the expected fraction of refOTUs with changes in relaxation time constants between the two antibiotic treatment intervals. The intra-ecosystem signature diversity (SD2) score was ≈8–20 expected equivalent signatures per 100 refOTUs (Figure 3C), indicating that many micro-organisms within each host ecosystem exhibited similar responses to the antibiotic treatments. Stated another way, a typical refOTU in a subject's gut ecosystems shared a similar response with ≈5–12 other refOTUs.

These analyses indicated that subject E's gut microbiota exhibited fewer long-term shifts in abundance levels and responded overall more uniformly to the antibiotic exposures. That is, subject E had significantly lower intra-signature and intra-ecosystem Signature Diversity scores, with an SD1_μ_score of 50% and SD2 score of 10, as compared with the other two subjects with SD1_μ_ scores >70% and SD2 scores ≈20. This differential behavior of subject E's microbiota was not discernible in the original analysis performed by Dethlefsen *et al.*, as they did not use techniques that quantified diversity of temporal responses. Our Signature Diversity analysis thus provides additional information about the functional diversity of subject E's microbiota, suggesting that this subject may have harbored a more ciprofloxacin-resistant flora prior to the experiments. Of note, subjects in the study had not received antibiotics in the past year before the experiments, but their antibiotic exposure history prior to this point was unknown.

The inter-ecosystem signature diversity (SD3) score for the 3 subjects was 48%(*p*-value<10^−6^using a permutation test with null hypothesis of independent ecosystems), indicating that there were substantial similarities in the time-dependent responses of the subjects' microbiota to the antibiotic treatments. As shown in Figure 3D, the SD3 score is a ratio of two SD2 scores: (1) SD2^D^, which is computed on a hypothetical combined ecosystem, in which refOTUs from different subjects probabilistically share prototype signatures, and (2) SD2^I^, which is a weighted average of independent SD2 scores computed separately on each subject. The SD3 score of 48% indicates that approximately as many prototype signatures were shared among the subjects as were unique to each subject. Thus, although subjects' microbiota did exhibit varied responses to the antibiotic treatments, as reported in the Dethlefsen *et al.* study, our findings indicate that there were substantial commonalities among responses. These commonalities could not have been found using the analysis techniques of the original study, which relied on calculations at individual time-points, in part because different sampling times for each subject made point-wise comparisons impossible. In contrast, MC-TIMME uses a continuous-time model of dynamics that leverages information from multiple time-points to estimate key dynamical properties, allowing comparisons across subjects on a common time-scale.

### Relaxation Time Distributions and Consensus Signature Groups

**Figure 4:**
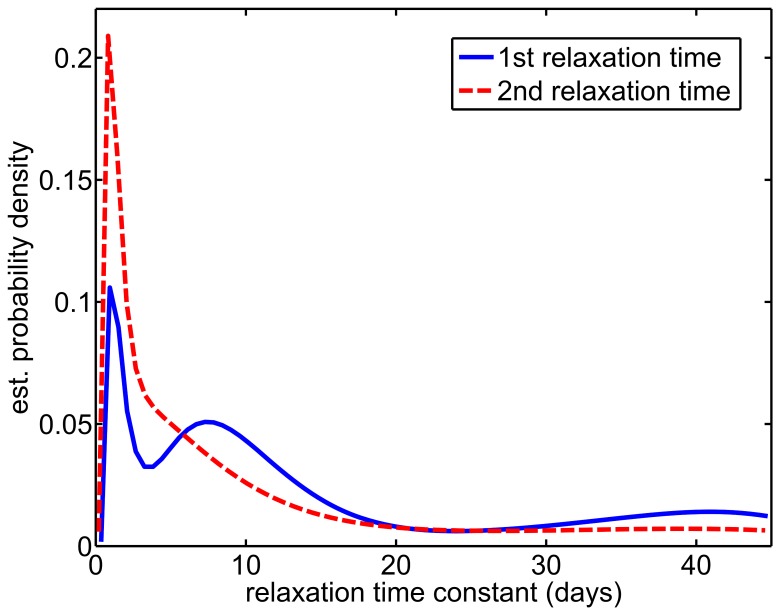
Relaxation Time Distributions for responses of human gut commensals to repeated antibiotic exposures. Each relaxation time constant characterizes the time for a reference operational taxonomic unit (refOTU) to reach an equilibrium relative abundance level in the ecosystem after an antibiotic pulse. Probability density functions were estimated for either the first post-antibiotic exposure interval (solid blue line, “1^st^ relaxation time”) or the second post-antibiotic exposure interval (dashed red line, “2^nd^ relaxation time”). A smoothing kernel algorithm was used to estimate probability density functions, using relaxation time constants from refOTUs from all subjects (756 time constants for each post-antibiotic exposure interval).

We generated Relaxation Time Distribution (RTD)plots using data from all three subjects, to investigate common trends in the rates at which the microbiota attained equilibrium levels after repeated antibiotic exposures (Figure 4). These plots depict estimated smoothed probability distributions of relaxation time constants, in units of days, for all refOTUs across all subjects. As shown in Figure 4, the Relaxation Time Distribution for the first post-antibiotic exposure interval is multi-modal. A large peak in the distribution at ≈1–3 days indicates that many refOTUs quickly reached equilibrium levels, while a subsequent broader peak suggests waves of microbial succession events among subpopulations that took longer to equilibrate. Interestingly, after the second antibiotic exposure, the relaxation time distribution became simpler and more concentrated, with more refOTUs exhibiting relaxation times around ≈1–3 days. This finding suggests that the first antibiotic treatment shifted gut ecosystems toward more rapidly equilibrating states, possibly by selecting for more antibiotic resistant organisms or for sub-communities that more quickly and stably established themselves in relevant niches.

To further our understanding of the differential responses of microbial sub-communities to antibiotic exposures, we next generated Consensus Signature Groups (CSGs), which represent groups of refOTUs that consistently covary in terms of relative abundances over time. Combining data from all subjects, MC-TIMME identified 125 CSGs. Interestingly, many of the CSGs contained refOTUs that are phylogenetically related or are common to all subjects. To assess the phylogentic relationships among refOTUs within each CSG, we calculated an enrichment *p*-value for taxonomic labels at the order, family and genus levels, based on a hypergeometric distribution null hypothesis. Approximately 61% of refOTUs belonged to CSGs significantly enriched for at least one taxonomic label at these levels, with most such CSGs shared across subjects. These results provide evidence that MC-TIMME detected biologically relevant sub-communities of organisms based only on evaluation of time-varying behaviors of refOTUs. MC-TIMME additionally discovered refOTUs that exhibited consistent behavior across all subjects: 88refOTUs were present in all 3 subjects, and of these 88 refOTUs, ≈25% were assigned to common CSGs. These sets of refOTUs, which consistently covary across all subjects, could serve as candidate biomarkers in future studies of antibiotic treatments or other perturbations to the gut flora.

**Figure 5:**
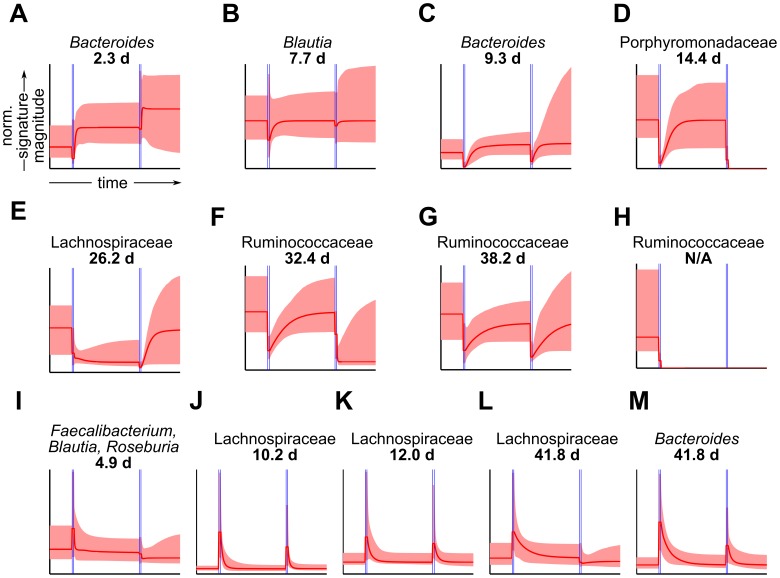
Consensus Signature Groups of human gut commensals ordered by relaxation time after first antibiotic pulse. Each panel (**A–M**) depicts a Consensus Signature Group (CSG), with signatures conformed to a common time-scale and amplitude to facilitate comparison across subjects and CSGs. Displayed CSGs are those containing at least one reference operational taxonomic units (refOTUs) shared among all subjects, and significantly enriched for at least one taxonomic label at the family or genus level (false discovery rate <0.05, hypergeometric tests). The horizontal axis indicates time in days and the vertical axis indicates normalized signature amplitude. Red dashed lines depict median inferred signatures, and shaded red areas indicate 95% credible intervals. Horizontal blue lines depict antibiotic exposure windows. Numbers above plots indicate relaxation time constants in units of days.(**A–H**) are CSGs showing decreases in relative abundance during the first antibiotic pulse, and (**I–M**) are CSGs showing increases in relative abundance during the first antibiotic pulse.

We created a time-line of the largest and best taxonomically defined Consensus Signature Groups (Figure 5A–M), to gain insight into the specific responses and successive equilibration of gut commensal sub-populations after antibiotic exposures. For purposes of visualization, we ordered the CSGs according to their relaxation time constants after the first antibiotic pulse, as these relaxation times exhibited the most variation. To facilitate interpretation, we included only CSGs containing at least one refOTU shared among all subjects, and significantly enriched (false discovery rate<0.05, hypergeometric tests)for at least one taxonomic label at the family or genus level. These criteria yielded 13 CSGs, containing ≈50% of all refOTUs. Of the 13 CSGs, 8 exhibited decreases in relative abundance during the first antibiotic pulse (Figure 5A–H), and 5 respectively exhibited increases (Figure 5I–M).

Among Consensus Signature Groups showing decreases in relative abundance during the first antibiotic pulse, those containing refOTUs in the genus *Bacteroides* (Figure 5A,C), or refOTUs in the related family Porphyromonadaceae (Figure 5D), were among the first groups to equilibrate after cessation of antibiotics. These groups of refOTUs had relaxation times <≈2 weeks, and returned to the same or higher relative abundances as compared to those prior to the first antibiotic pulse. The *Bacteroides* [51]are obligate anaerobes. Members of this genus, in particular *B. fragilis*, are known to have greater resistance to ciprofloxacin, mediated in part by several bacterial factors, coupled with reduced activity of the antibiotic under anaerobic conditions [40], [52]. The *Bacteroides* can also be opportunistic pathogens and are capable of developing resistance to multiple classes of antibiotics after repeated exposures [53], [54]. These characteristics may explain why the *Bacteroides* were among the first genera found by MC-TIMME to recover post-antibiotic treatment. Interestingly, another CSG significantly enriched for refOTUs of genus *Bacteroides* (Figure 5M) exhibited an increase in relative abundance during antibiotic treatment, with a very slow return to equilibrium levels (relaxation time ≈42 days). This finding suggests that subjects consistently harbored antibiotic resistant *Bacteroides*, echoing concerns that members of this genus could serve as reservoirs of resistance genes for more frankly pathogenic bacteria [53], [54].

MC-TIMME also identified another quickly equilibrating sub-community that contained refOTUs belonging to acetate [55] and butyrate [56] producing genera (Figure 5I). This sub-community showed increases in relative abundance during antibiotic treatments and was significantly enriched for refOTUs belonging to the genera *Blautia*, *Faecalibacterium*, or *Roseburia*. Many *Blautia* species are acetogens, producing acetate from H_2_ and CO_2_ through the acetyl-CoA pathway [55]. Acetate has known downstream effects on the microbial production of butyrate [57]. Butyrate, a 4-carbon short chain fatty acid, has important roles in maintaining colonic health of the host, providing a luminal source of energy to the epithelial barrier [57], while limiting the degree of autophagy in host colonocytes and reducing the host's susceptibility to agents that might otherwise promote damage to the colonic mucosa [58]. Members of the genera *Faecalibacterium* and *Roseburia* are prominent butyrate producers in the human gut [56]. Our CSG analysis suggests that the identified *Blautia*, *Faecalibacterium*, and *Roseburia* refOTUs may operate as a functional multi-species community in the gut, one demonstrating relative resilience tociprofloxacin's effects. This finding highlights MC-TIMME's ability to identify and track over time and across multiple subjects, bacterial sub-communities with potential benefits to the host. Interestingly, MC-TIMME discovered a second CSG containing *Blautia* refOTUs, but not the butyrate producing genera (Figure 5B),and that exhibited a different response pattern, with a decrease in relative abundance during the first antibiotic pulse, and relatively rapid return to pre-antibiotic relative abundances. Of note, certain *Blautia* species, such as *B. hydrogenotrophica*, use a broader range of substrates for acetogenesis than other species in the genus [55].The presence of such *Blautia* species with greater metabolic flexibility in the CSG depicted in Figure 5B could explain the lack of butyrate producers in this consensus signature group.

Several Consensus Signature Groups contained refOTUs belonging to the family Ruminococcaceae (Figure 5F,G,H). These CSGs showed decreases in relative abundance during the first antibiotic pulse and equilibrated slowly thereafter. In fact, one group of these organisms (Figure 5H), become undetectable after the first antibiotic pulse, and another group (Figure 5F) declined to very low relative abundance levels after the second pulse. The Ruminococcaceae are overall obligately anaerobic, fastidious organisms that may require substrates produced as by-products of metabolism by earlier colonizers in gut luminal food-webs [57]–[59]. Thus, the delay in which these consensus groups of Ruminococcaceae recovered may be due to high degrees of dependence on activities of other organisms in the ecosystem.

MC-TIMME also discovered a number of Consensus Signature Groups containing refOTUs from the family Lachnospiraceae (Figure 5E,J,K,L). The majority of these CSGs showed increases during the antibiotic pulses, with fairly long relaxation times to pre-antibiotic relative abundance levels. The Lachnospiraceae are a large family of difficult to cultivate organisms, some of which have been found in close association with the mucous layer over the distal colonic epithelium [60]. Although little is known about the antibiotic susceptibilities of these organisms, it has been hypothesized that they may have evolved special mechanisms to survive the higher concentrations of endogenously produced host anti-microbial peptides present in this niche [60]. Our CSG analysis identified distinct groups of refOTUs from the Lachnospiraceae family that exhibited prolonged increases in relative abundance after ciprofloxacin exposure. These findings provide new information about this poorly understood bacterial family, which could be used to guide future studies to evaluate potential mechanisms of innate antibiotic resistance among Lachnospiraceae sub-communities.

### Automated Experimental Design

Application of metagenomic techniques to diagnostic medicine will require human clinical trials across many subjects to ascertain time-dependent effects and responses of the microbiota relative to a defined perturbation or clinical course of disease. The complicated logistics and expense of such trials highlight the need for computational techniques to optimize sampling across subjects.

We developed an algorithm for automated experimental design, and applied it to the Dethlefsen *et al.* dataset to explore how the experimental design of a longitudinal study of the microbiota could be improved for future, larger trials. Our algorithm uses the data from a set of previously performed experiments to estimate an initial model of prototype signatures. This initial model is used to find a set of time-points that maximize the information that can be gained from future, hypothetical experiments. In general, our algorithm prioritizes time-points for sampling in future experiments around time-points in the original experiment that had the highest degree of uncertainty in the model. The selected time-points may differ from those in the original experiments, and thus indicate when increased sampling could better estimate dynamics of the ecosystems under study, or when reduced sampling still yields sufficient information.

**Figure 6:**
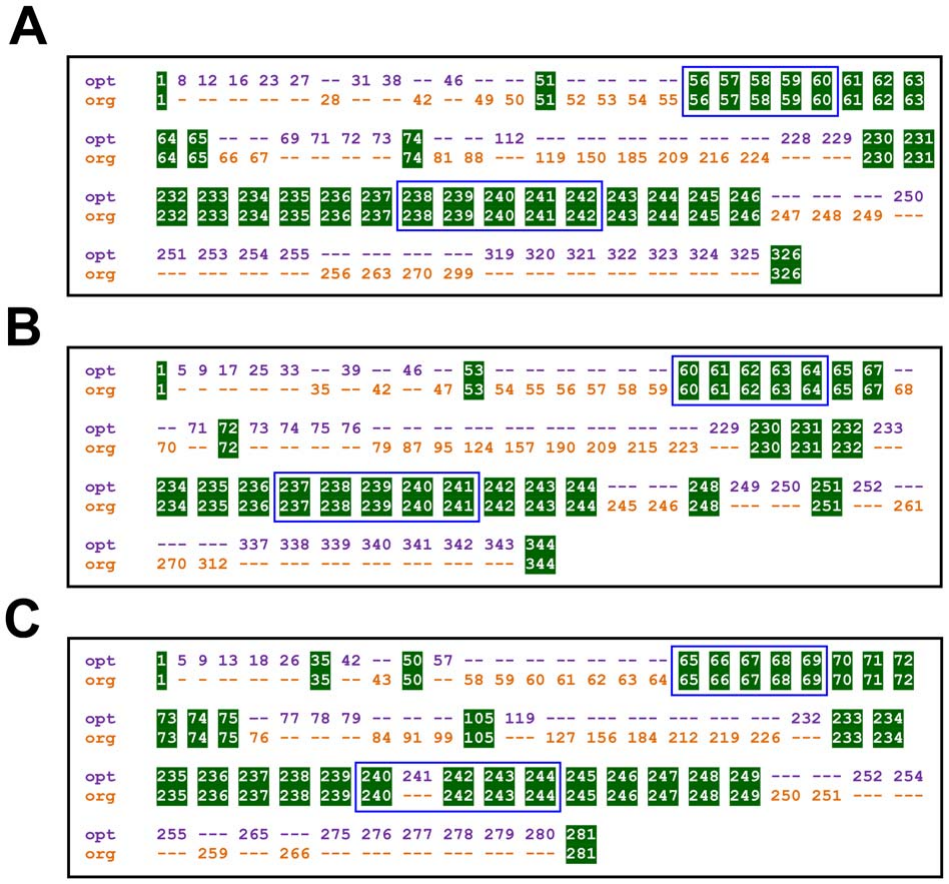
Optimized experimental design for studying responses of the human gut microbiota to repeated antibiotic exposures. Each panel (**A–C**) depicts an optimized design for one subject in the study. (**A**) Subject D. (**B**) Subject E. (**C**) Subject F. The rows in each panel depict the time-points from the optimal (“opt”) and original (“org”) designs. Times are in days from the start of the experiments. Time-points that overlap in both designs are shown in green boxes. Time-points that are unique to either of the optimal or original designs are depicted with colored numbers (optimal = purple, original = orange). The two antibiotic exposure intervals are shown as blue rectangles.

Figure 6 depicts the optimized experimental design produced by our algorithm for each subject. To facilitate comparisons among designs, we restricted our algorithm to choose the same numbers of time points as were used in the original design. Our algorithm generated an optimal design that consistently differed from the original design, over certain temporal intervals. On the pre-antibiotic interval, the optimized design required more uniform sampling, reflecting the modeling assumption that host ecosystems were at equilibrium prior to antibiotic exposure. For the period immediately after the first antibiotic exposure, the optimized design required additional frequent sampling beyond the one week of the original design. This increased sampling requirement is consistent with the relaxation time distribution on the first post-antibiotic treatment interval shown in Figure 4, which indicates the presence of transient effects beyond one week for many refOTUs. However, the optimal design required fewer subsequent samples on the first post-antibiotic treatment interval, suggesting that MC-TIMME can effectively leverage earlier time-points obtained while the ecosystem is still equilibrating to estimate later behavior near steady-state. During the period immediately after the second antibiotic exposure, the optimal design generally required less frequent sampling than did the original design. This reduced sampling requirement reflected the shorter relaxation time distribution on the second post-antibiotic interval as shown in Figure 4. Finally, the optimal design required considerably more sampling at the end of the time-series; the original experiments clearly under-sampled after the second antibiotic exposure, as is evident from high variability in model parameter estimates on this interval (see Figure 5 and Figure S2).

To assess the predictive accuracy of our experimental design algorithm, we evaluated its ability to find a set of experiments to best estimate a model to predict held-out data (Figure S3). We used root mean square error (RMSE) to measure predictive accuracy. RMSE is the square root of the sum of squared differences between actual and predicted sequencing counts, averaged over refOTUs and time-points. Evaluation of predictive accuracy is important to assess the degree to which a probabilistic model generalizes to new data, without over-fitting features particular to one dataset. To perform this evaluation, we estimated optimal experimental designs for each subject, using three design strategies. For the first strategy, a sequential design, we gave the experimental design algorithm data for all refOTUs observed at a subset of time-points, and asked the algorithm to estimate additional time-points to sample in the *same* subject. For the second strategy, a cross-subject design, we gave the experimental design algorithm all observed data from one subject, and asked the algorithm to estimate time-points to sample for a *different* subject. In the third strategy, a dispersed design, we did not use the experimental design algorithm, and simply chose time-points to sample that were as evenly spaced on the study interval as possible. The dispersed design uses no information from observed data, and thus served as a baseline with which to compare the other two design strategies.

The two experimental design strategies (sequential and cross-subject)that use prior information improved on the uninformative dispersed strategy by an average of 13%, as measured by reduction in prediction accuracy (RMSE). Of the two informative strategies, neither consistently dominated the other. However, the cross-subject strategy did substantially outperform the sequential strategy for subject D. This subject exhibited the highest Signature Diversity equilibrium level (SD1_μ_) score, meaning that many refOTUs in this subject changed equilibrium levels subsequent to one or both antibiotic exposures. Consequently, equilibrium levels for refOTUs in subject D were harder to predict from prior equilibrium levels. Thus, the sequential design strategy, which uses only partial time-series data as input to the design algorithm, suffered in performance. In contrast, the cross-subject strategy that uses complete data from another subject, performed particularly well for subject D, because it leveraged prototype signatures predicted from subject E or F that were substantially similar to those in subject D.

## Discussion

We presented MC-TIMME, a unified computational framework for inferring dynamic signatures of the microbiota from high-throughput sequencing time-series datasets, and applied our framework to discover new features of the *in vivo* response of human gut microbes to antibiotic treatments. Our work represents both biologically and computationally significant advances. From the biological perspective, our study provides new insights into the differential and dynamic effects of antibiotic treatments on commensal bacteria in the human gut. Antibiotics disrupt the commensal flora, which can contribute to overgrowth and pathogenic effects of organisms such as *Clostridium difficile*, the cause of pseudomembranous colitis [61]. However, antibiotic effects have primarily been studied in pathogens; the range of effects on complex commensal populations remains largely unknown. Our results provide evidence, consistent across multiple human subjects, that sub-groups of commensals exhibit distinct temporal responses to treatment with a broad spectrum antibiotic. These results illustrate the staged dynamics of responses among sub-populations of commensals, and will enable future experimental studies to characterize the underlying molecular mechanisms behind these differential responses. From the computational standpoint, our study provides a robust, general-purpose framework for extracting fundamental information on ecosystem dynamics from massive sequencing datasets. Our framework may be applied to types of data other than 16S phylotypes, such as metagenomics or RNA Seq data, as well as model systems in animal or plant hosts, or studies in soil or marine environments. MC-TIMME employs probabilistic models of dynamics and associated measures of their properties, which yield important functional information that standard techniques for analyzing microbial communities cannot. Our use of adaptive Bayesian methods not only increases the strength of statistical inferences, but also provides signatures of microbial responses that are robust across multiple experimental subjects. Additionally, MC-TIMME enables optimal design of new time-series experiments, which will provide a strong foundation for future longitudinal studies of the microbiota.

Our results on automated experimental design strategies have implications for how future longitudinal studies of the microbiota should be designed. An automated cross-subject design strategy generally performed comparably to or better than a sequential design strategy. A cross-subject design strategy uses all data from one subject to predict a future experimental design for a second subject. In contrast, a sequential design strategy uses limited samples from one subject to predict a future experimental design for the same subject. In the past, when the costs of experimentally interrogating samples were high, strategies using automated design were advocated in which samples would be over-collected, frozen, and then sequential design methods would be used to select the next samples to interrogate [24]. Our findings suggest that, in an era of plummeting sequencing costs, automated experimental designs based on pilot studies with small cohorts may prove more effective, particularly for clinical trials in which sample collection costs and logistics can be the limiting factors. However, confirmation of this hypothesis will require larger numbers of subjects and more detailed information about the heterogeneity of cohorts, with respect to factors such as demographics and environmental exposures. Additionally, we used a general-purpose, information theoretic utility function as a basis for selecting optimal experimental designs. This utility function has proven useful in many prior studies (see e.g., [46]), and performed well in our analyses. However, our framework for experimental design could readily be extended, by using utility functions that explicitly include financial or other costs involved in performing experiments.

Analysis of host microbial ecosystems solely by 16S phylotyping has inherent limitations. Sequencing based methods suffer from various biases, due to factors such as the DNA extraction method and sequencing platform utilized [62]. Additionally, from 16S phylotyping data, it is only possible to infer abundances of taxa relative to other members of the microbial ecosystem detected with sequencing, and not the actual biomasses of individual taxa relative to the input mass of material analyzed. Thus, from our relaxation time analysis, one cannot infer the time required for taxa to equilibrate in terms of their absolute biomasses *in vivo*. Nonetheless, many studies have shown that relative abundances of organisms serve as important ecological indicators [2]. Relative abundances reflect differential abilities of organisms to compete for and effectively use limited resources, and thus provide insights into the roles of sub-communities within larger host ecosystems. However, the most profound limitation to 16S phylotyping data is that it is only useful for identifying which bacteria are present, not what they are doing. Ultimately, targeted or high-throughput functional studies [63] are essential for following up hypotheses generated based on 16S phylotyping.

MC-TIMME can be extended with alternate models of dynamics for analyzing other time-series datasets. The key components of the model, including the infinite mixture model for prototype signatures and the noise model for counts data, employ general-purpose inference techniques that would not need to be modified to accommodate different models of dynamics. However, the Reversible Jump MCMC techniques we used for inference of intra-signature dimensionality changes require model-specific moves; in future work, more general techniques such as sparse priors [64], [65]could be employed for inference of this portion of the model. For the experimental system we modeled, with defined antibiotic administrations, we assumed that perturbations to the microbiota start at known time-points. This model of dynamics could be extended for analyzing observational studies, in which naturally occurring perturbations may occur, by adding latent variables that automatically determine switch-points in dynamics. Another direction for extension would be building more elaborate relaxation time models. We used a relaxation time model based on ordinary differential equations, which assumes instantaneous transitions to different dynamic regimes and monotonic exponential decay to equilibrium levels. A more detailed model could allow smooth transitions between regimes, and richer kinetics of decay to equilibrium, to capture more subtle or chronic responses to perturbations. Additionally, more complex temporal correlations could be captured using a stochastic differential equation model such as the Ornstein-Uhlenbeck process [66]. Finally, longitudinal covariates, such as subject diet, could be added to the model of dynamics to capture exogenous factors that may affect the microbiota. Such extensions to MC-TIMME will enable increasingly sophisticated longitudinal studies, to expand our knowledge of the role of the microbiota in human health or disease.

## Supporting Information

Figure S1 **Model of dynamics for prototype signatures.** An example of a prototype signature (solid blue line) is depicted. The model of dynamics for each prototype signature is a function continuous in both time and values, which is used to parameterize the mean of the negative binomial distribution (NBD). The function is defined piece-wise on 5 intervals: (A) pre-antibiotic exposure, (B) first antibiotic pulse, (C) first post-antibiotic exposure, (D) second antibiotic pulse, and (E) second post-antibiotic exposure. The function is constant on intervals A, B and D. On intervals C and E, the function follows an exponential relaxation process with initial value *X*, equilibrium value *μ*, and relaxation time constant *λ*; an equation for the corresponding relaxation process is shown above intervals C and E. Equilibrium levels for each interval are depicted at the right of the figure.
(TIF)

Figure S2 **Representative individual signatures of human gut commensals perturbed by antibiotic pulses.** Panels (**A–F**) depict normalized individual signatures for reference operational taxonomic units (refOTUs) from a single human subject. The vertical axis represents sequencing counts per 10,000 total reads normalized across experiments. The horizontal axis represents time in days since the start of the experiment. Vertical blue lines depict the two windows of antibiotic exposure (56 to 60 and 238 to 242 days). Dashed red lines depict median inferred individual signatures, and shaded red areas depict 95% credible intervals. Each refOTU is labeled with its number from the original dataset and its taxonomic assignment at the family and genus levels. The symbol *λ_c_* denotes the inferred median relaxation time constant on the first post-antibiotic interval, and *λ_e_* denotes the corresponding relaxation time constant on the second post-antibiotic interval. The symbols *μ_a_*, *μ_c_*, and *μ_e_* denote the equilibrium levels on pre- and first and second post-antibiotic intervals. The five probabilities shown indicate the probability that: 1) the relaxation times on both post-antibiotic intervals are equal, and 2–5) the three equilibrium levels on the pre- and first and second post-antibiotic intervals have a particular pattern.
(TIF)

Figure S3 **Predictive performance of the experimental design algorithm using different design strategies.** Predictive performance was evaluated on held-out time-points, with accuracy assessed using root mean square error (RMSE). (**A**) The sequential design strategy used data for all reference operational taxonomic units (refOTUs) observed at a subset of time-points in a subject, to estimate additional time-points to sample in the same subject. (**B**) The cross-subject design strategy used all observed data from refOTUs in one subject, and estimated time-points to sample in a different subject. A dispersed design strategy was used as a baseline for comparison. The dispersed design did not use the experimental design algorithm, and chose time-points to sample that were as evenly spaced on the study interval as possible.
(TIF)

Protocol S1 **Detailed methods.**
(PDF)
