# Supplementary figures and images for: Inferring Dynamic Signatures of Microbes in Complex Host Ecosystems
Source: PLoS Comput Biol. 2012 Aug 2;8(8):e1002624. doi: 10.1371/journal.pcbi.1002624 (PMC3410865; doi:10.1371/journal.pcbi.1002624)

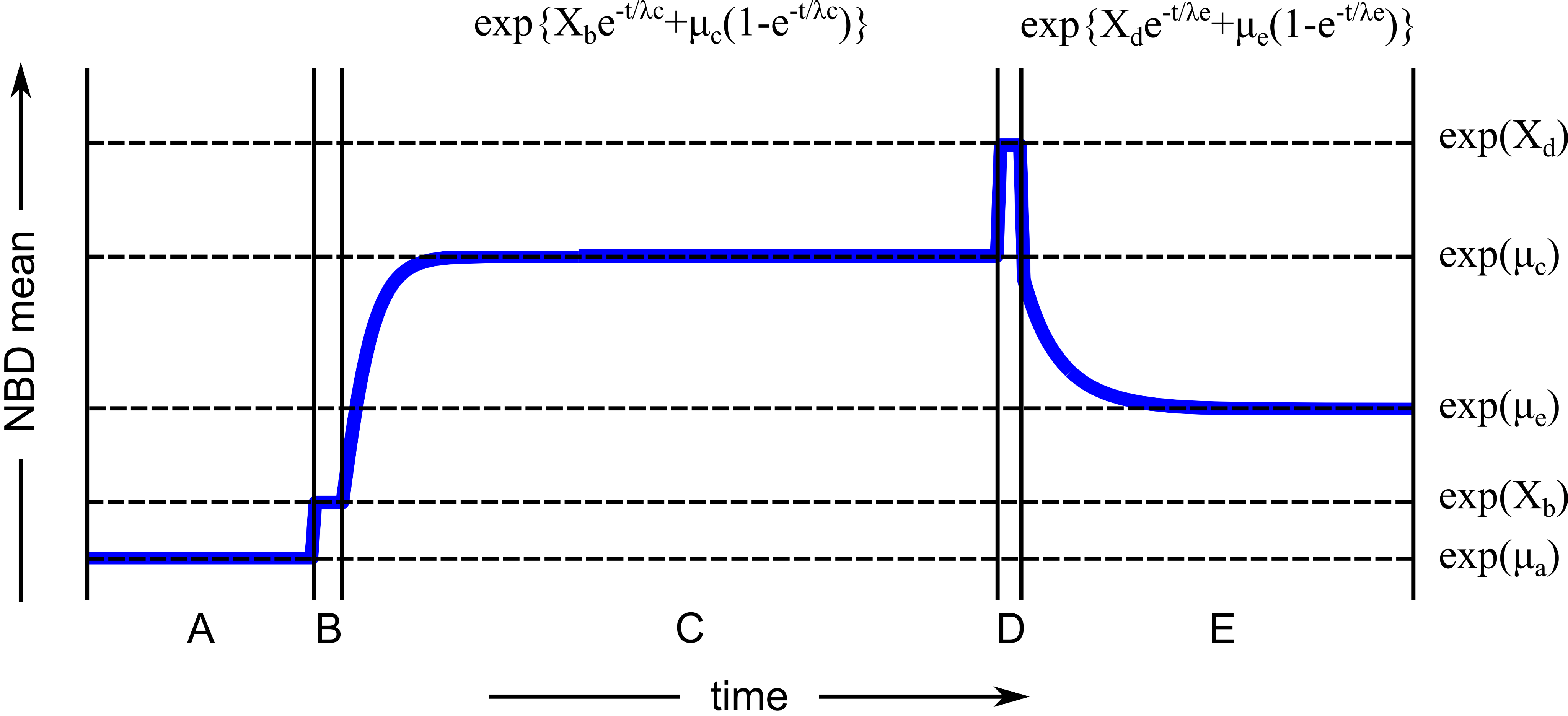

Supplement: Figure S1 — Model of dynamics for prototype signatures. An example of a prototype signature (solid blue line) is depicted. The model of dynamics for each prototype signature is a function continuous in both time and values, which is used to parameterize the mean of the negative binomial distribution (NBD). The function is defined piece-wise on 5 intervals: (A) pre-antibiotic exposure, (B) first antibiotic pulse, (C) first post-antibiotic exposure, (D) second antibiotic pulse, and (E) second post-antibiotic exposure. The function is constant on intervals A, B and D. On intervals C and E, the function follows an exponential relaxation process with initial value X, equilibrium value μ, and relaxation time constant λ; an equation for the corresponding relaxation process is shown above intervals C and E. Equilibrium levels for each interval are depicted at the right of the figure. (TIF) [file pcbi.1002624.s001.tif]

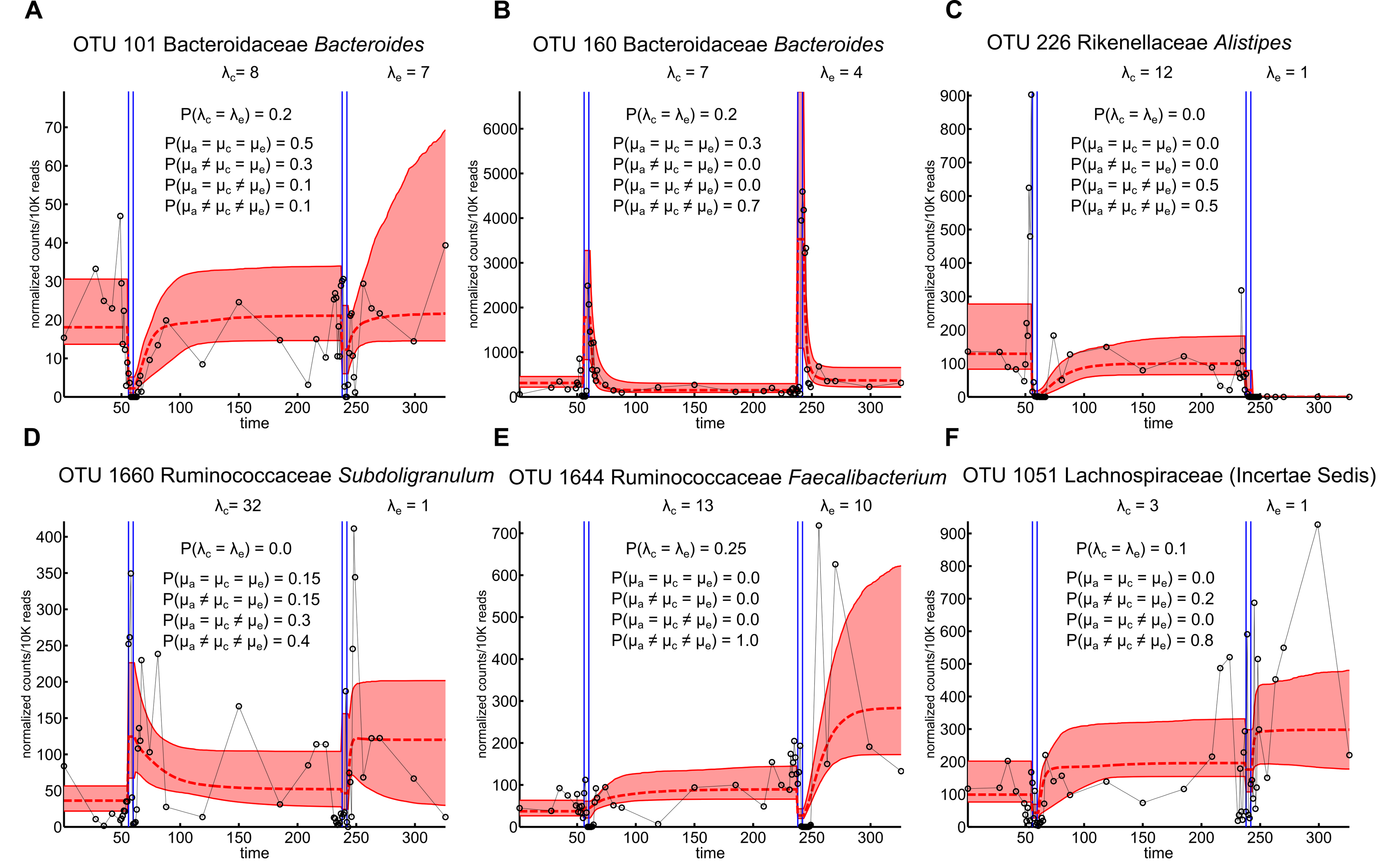

Supplement: Figure S2 — Representative individual signatures of human gut commensals perturbed by antibiotic pulses. Panels (A–F) depict normalized individual signatures for reference operational taxonomic units (refOTUs) from a single human subject. The vertical axis represents sequencing counts per 10,000 total reads normalized across experiments. The horizontal axis represents time in days since the start of the experiment. Vertical blue lines depict the two windows of antibiotic exposure (56 to 60 and 238 to 242 days). Dashed red lines depict median inferred individual signatures, and shaded red areas depict 95% credible intervals. Each refOTU is labeled with its number from the original dataset and its taxonomic assignment at the family and genus levels. The symbol λc denotes the inferred median relaxation time constant on the first post-antibiotic interval, and λe denotes the corresponding relaxation time constant on the second post-antibiotic interval. The symbols μa, μc, and μe denote the equilibrium levels on pre- and first and second post-antibiotic intervals. The five probabilities shown indicate the probability that: 1) the relaxation times on both post-antibiotic intervals are equal, and 2–5) the three equilibrium levels on the pre- and first and second post-antibiotic intervals have a particular pattern. (TIF) [file pcbi.1002624.s002.tif]

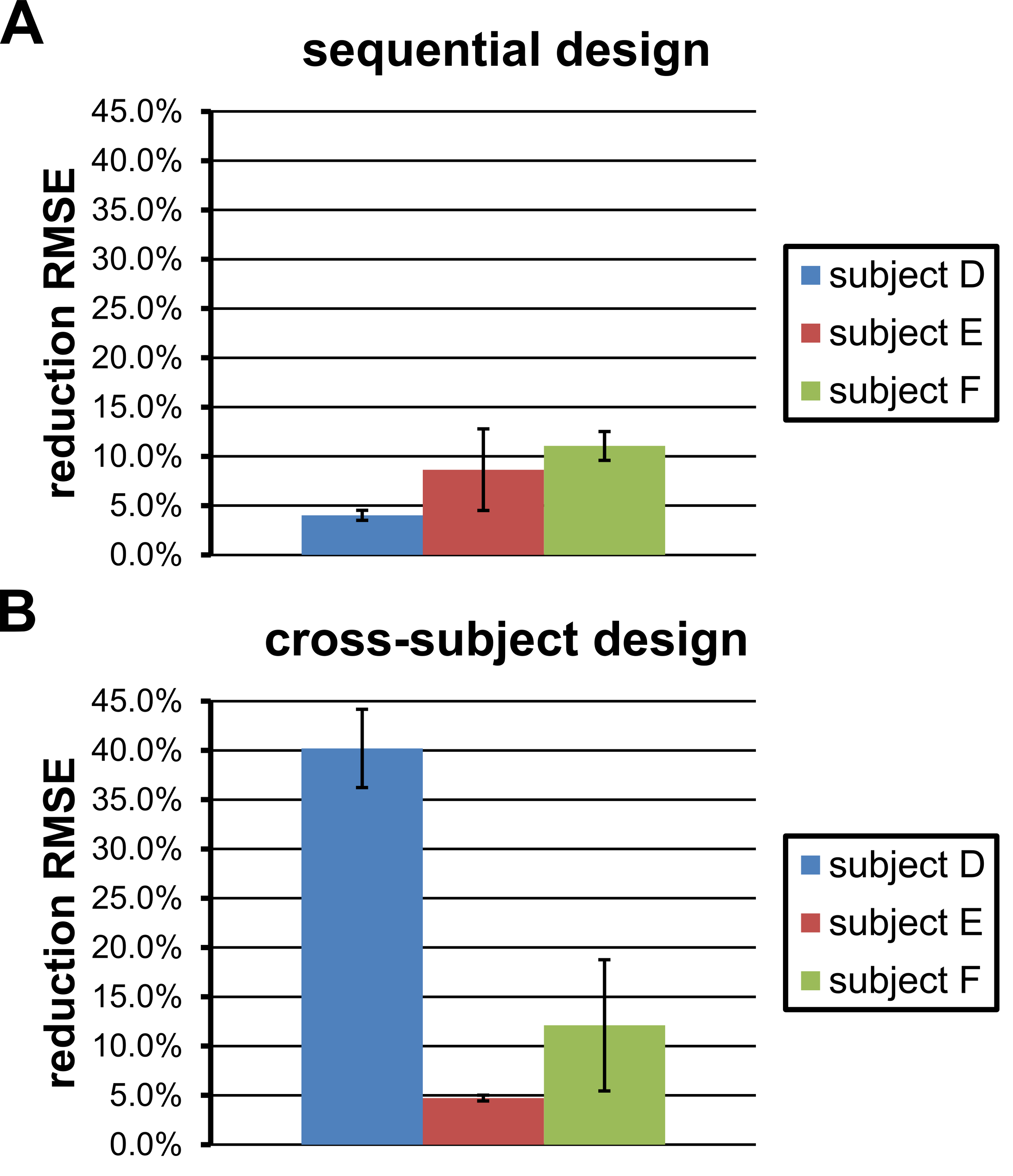

Supplement: Figure S3 — Predictive performance of the experimental design algorithm using different design strategies. Predictive performance was evaluated on held-out time-points, with accuracy assessed using root mean square error (RMSE). (A) The sequential design strategy used data for all reference operational taxonomic units (refOTUs) observed at a subset of time-points in a subject, to estimate additional time-points to sample in the same subject. (B) The cross-subject design strategy used all observed data from refOTUs in one subject, and estimated time-points to sample in a different subject. A dispersed design strategy was used as a baseline for comparison. The dispersed design did not use the experimental design algorithm, and chose time-points to sample that were as evenly spaced on the study interval as possible. (TIF) [file pcbi.1002624.s003.tif]
