# Supplementary material for: Inferring Dynamic Signatures of Microbes in Complex Host Ecosystems
Source: PLoS Comput Biol. 2012 Aug 2;8(8):e1002624. doi: 10.1371/journal.pcbi.1002624 (PMC3410865; doi:10.1371/journal.pcbi.1002624)
Supplement: Protocol S1 — Detailed methods. (PDF) [file pcbi.1002624.s004.pdf]

Protocol S1: Inferring Dynamic Signatures of Microbes in Complex Host Ecosystems

1 Overview

MC-TIMME is based on Dirichlet Process Infinite Mixture Models (DP IMMs), which are a class of non-parametric Bayesian models. DP IMMs can be derived as the limit of Finite Mixture Models (FMMs), which assume that data y arise from a weighted mixture of K probability distributions:

$$\mathrm{p}(\mathsf{y} \mid \boldsymbol{\theta}, \boldsymbol{\pi}) = \sum_{k=1}^{K} \pi_k \phi(\boldsymbol{y};\ \boldsymbol{\theta}_k)$$

Here, $\boldsymbol{\pi}$ are mixture weights (probabilities) such that $\sum_{k=1}^{K} \pi_k = 1$, $\boldsymbol{\theta}_k$ are the parameters for mixture component k, and $\phi(\cdot;\ \cdot)$ is a probability density function for the mixture components.

For the MC-TIMME model, the mixture components are the continuous-time and continuous-valued prototype signatures. Each prototype signature is characterized by a set of variables that specify the shape of the signature. The number of variables used to model the shape of each signature is adaptively learned by MC-TIMME. Additionally, latent (hidden) variables are used to assign the observed sets of sequencing counts to prototype signatures.

Figure 1 depicts the MC-TIMME generative model structure in graphical model form with plate notation. The model is hierarchical, with variables at higher levels parameterizing prior distributions for variables at lower levels.

The data generation model is as follows. Assume that we have observed counts of sequencing reads for O_s refOTUs in S subjects at T_s time-points, with each data point denoted by y_{sot}. Prototype signatures are associated with mixture weights $\boldsymbol{\pi}$ and parameters $\boldsymbol{\Theta}$. Each refOTU o in subject s is assigned to a prototype signature via a variable $\mathsf{z}_{so} \mid \boldsymbol{\pi} \sim \text{Multinomial}(\boldsymbol{\pi})$. For a given prototype signature k, $\boldsymbol{\theta}_k$ is a parameter vector specifying a continuous-time and continuous-valued function, as described in Section 3. We assume that the conditional distribution for the observation y_{sot} is the Negative Binomial Distribution (NBD), with mean parameterized by the function $f(t, \boldsymbol{\theta}_k)$, and variance controlled by parameters $\boldsymbol{\epsilon}$:

$$\mathsf{y}_{sot} \mid \mathsf{z}_{so} = k, \boldsymbol{\theta}_k \sim \text{Neg-Bin}(e^{f(t,\boldsymbol{\theta}_k)+\gamma_{so}+\psi_{st}}, \boldsymbol{\epsilon}) \tag{1}$$

Here, γ_{so} and ψ_{st} are offset terms that specialize the underlying prototype signature to an individual signature for refOTU o in subject s (see Section 2).

Thus, the data is assumed to be generated via a conditional Generalized Linear Model (GLM) using the Negative Binomial Distribution, with parameterization dependent on the prototype signature to which the data is assigned.

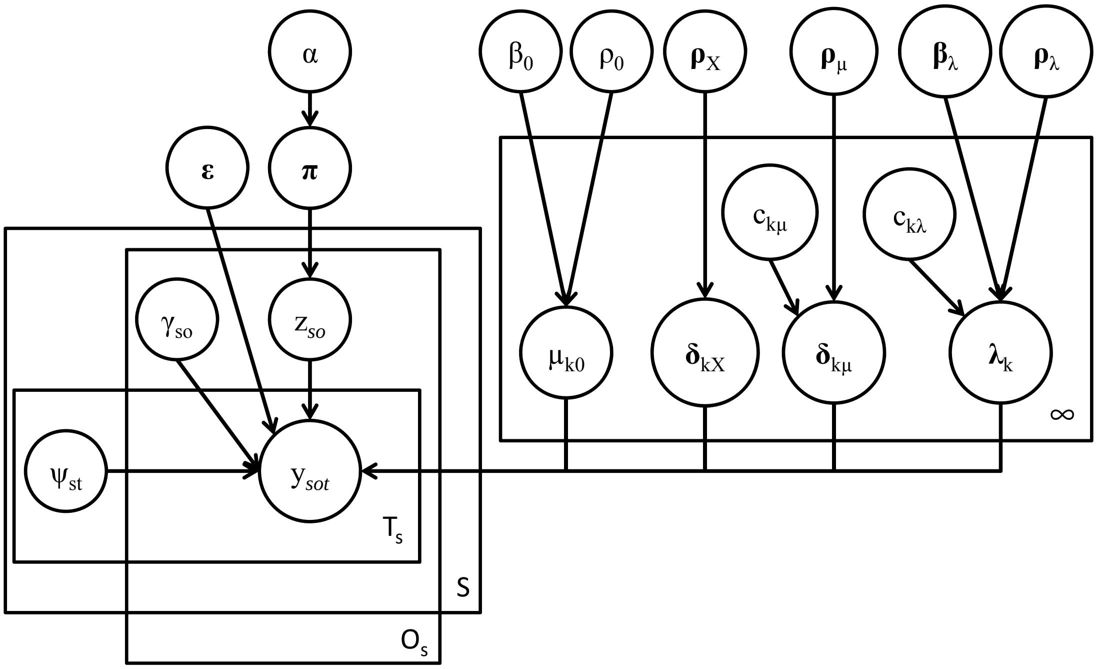

Figure 1: The MC-TIMME model depicted in graphical model form. Circles represent random variables and edges represent dependencies. Rectangles (plates) represent repeated variables. For example, the variable y_{sot} represents the observed counts for refOTU o in subject s at time-point t. This variable lies within three overlapping plates, which represent repetition over S subjects, O_s refOTUs, and T_s time-points. Arrows pointing into y_{sot} indicate that this variable depends directly on several others. The variable z_{so} represents a probabilistic assignment of refOTU o in subject s to a particular *prototype signature*. The variables γ_{so} and ψ_{st} are offset terms that specialize the prototype signature to a refOTU specific *individual signature*. The vector of variables $\boldsymbol{\epsilon}$ controls the variability of observed data generated by each individual signature. The variables μ_{k0}, $\boldsymbol{\delta}_{kX}$, $\boldsymbol{\delta}_{k\mu}$ and $\boldsymbol{\lambda}_k$ specify the shape of prototype signature k. The variables $c_{k\mu}$ and $c_{k\lambda}$ control the dimensionality of the prototype signature (e.g., the number of active variables). The infinity sign in the corner of the plate surrounding these variables indicates that there are a potentially unlimited number of prototype signatures. Note that the model also uses an additional level of hyperparameters for the prior distributions, which are not shown for clarity.

The mixture weights $\boldsymbol{\pi}$ are assumed to have a stick-breaking prior, $\boldsymbol{\pi} \sim \text{Stick}(\alpha)$, where α is a concentration parameter. The stick-breaking prior generates a countably infinite partition of the unit interval [0,1], with the "evenness" of the partition controlled by the concentration parameter α. The stick-breaking prior on $\boldsymbol{\pi}$ is defined constructively as:

$$\pi'_j \mid \alpha \sim \text{Beta}(1, \alpha)$$

$$\pi_j = \pi'_j \prod_{l=1}^{j-1} (1 - \pi'_l)$$

See [1] for further details regarding the stick-breaking prior formulation for the Dirichlet Process.

2 Signature offset terms

The term ψ_{st} specifies the offset from a prototype signature, specific to subject s at time-point t. This offset serves to standardize the samples to compensate for differing numbers of sequencing reads. This offset is estimated from data using the formula:

$$\psi_{st} = \log(\sum_o y_{sot}) - \text{median}_\tau \{\log(\sum_o y_{so\tau})\}$$

The term γ_{so} specifies the offset from a prototype signature, specific to refOTU o in subject s. This offset serves to standardize refOTU o according to its average abundance over time, and thus highlights the shape of the individual signature, rather than its absolute magnitude, for comparing individual signatures across refOTUs. This offset is estimated from data using the formula:

$$\gamma_{so} = \frac{\sum_t \log(y_{sot} + \iota) - \psi_{st}}{T_s}$$

Here, $\iota = 0.25$ to avoid taking the logarithm of zero.

3 Dynamical model for antibiotic pulse experiments

3.1 Specification

We assume that dynamics may differ over five intervals delimited by the antibiotic treatments in the experiments: (a) pre-antibiotic, (b) antibiotic treatment one, (c) post-antibiotic treatment one, (d) antibiotic treatment two, and (e) post-antibiotic treatment two. The main dynamics of interest, with regards to the extent of recovery or alterations in the commensal flora, occur on intervals (c) and (e).

We assume that dynamics for prototype signature k on interval (c), the period after the first antibiotic treatment, are specified by the following differential equation:

$$\dot{\eta}_{kc}(t) = \frac{1}{\lambda_{kc}} \{\mu_{kc} - \eta_{kc}(t)\}$$

Solving for $\eta_{kc}(t)$, we get:

$$\eta_{kc}(t) = f_c(t, X_{kb}, \mu_{kc}, \lambda_{kc}) = X_{kb} e^{-t/\lambda_{kc}} + \mu_{kc}(1 - e^{-t/\lambda_{kc}}) \tag{2}$$

The dynamical model on interval (c) thus specifies a relaxation process with initial value X_{kb} that reaches to equilibrium level μ_{kc} with relaxation time λ_{kc}.

For the second post-antibiotic exposure interval, (e), we assume a similar model but with initial value X_{kd}, equilibrium level μ_{ke}, and relaxation time λ_{ke}:

$$\eta_{ke}(t) = f_e(t, X_{kd}, \mu_{ke}, \lambda_{ke}) = X_{kd} e^{-t/\lambda_{ke}} + \mu_{ke}(1 - e^{-t/\lambda_{ke}}) \tag{3}$$

For the pre-antibiotic exposure interval (a), and the two antibiotic exposure intervals (b) and (d), we assume constant values for the equilibrium levels:

$$\eta_{ka}(t) = \mu_{ka}$$

$$\eta_{kb}(t) = X_{kb}$$

$$\eta_{kd}(t) = X_{kd}$$

For the parameter controlling NBD variance, we assume it is equal to ϵ_1 on intervals (a), (c) and (e), and equal to ϵ_2 on the antibiotic treatment intervals.

We assume the parameters of adjacent intervals are dependent via Gaussian random walks:

$$\mu_{ka} \sim \text{Normal}(\beta_0, \rho_0^2)$$

$$X_{kb} = \mu_{ka} + \delta_{k\mu_{a\to b}} \quad \text{s.t.} \quad \delta_{k\mu_{a\to b}} \sim \text{Normal}(0, \rho_X^2)$$

$$\mu_{kc} = \mu_{ka} + \delta_{k\mu_{a\to c}} \quad \text{s.t.} \quad \delta_{k\mu_{a\to c}} \sim \text{Normal}(0, \rho_\mu^2)$$

$$X_{kd} = \mu_{kc} + \delta_{k\mu_{c\to d}} \quad \text{s.t.} \quad \delta_{k\mu_{c\to d}} \sim \text{Normal}(0, \rho_X^2)$$

$$\mu_{ke} = \mu_{kc} + \delta_{k\mu_{c\to e}} \quad \text{s.t.} \quad \delta_{k\mu_{c\to e}} \sim \text{Normal}(0, \rho_\mu^2)$$

We specify the relaxation time parameters λ_{kc} and λ_{ke} as follows. To reflect the time-scale of the experiments, it's desirable to constrain these parameters within specified ranges. This is accomplished using softmax functions:

$$\lambda_{kc} = \frac{1}{1 + e^{-\xi_{kc}}} \{\lambda_{\text{max}} - \lambda_{\text{min}}\} + \lambda_{\text{min}}$$

$$\xi_{kc} \sim \text{Normal}(\beta_{\lambda_c}, \rho_{\lambda c}^2)$$

Here, λ_{max} and λ_{min} specify maximum and minimum values for the relaxation time parameters.

We assume that λ_{kc} and λ_{ke} are depedent via a Gaussian random walk:

$$\lambda_{ke} = \frac{1}{1 + e^{-(\xi_{kc} + \xi_{ke})}} \{\lambda_{\text{max}} - \lambda_{\text{min}}\} + \lambda_{\text{min}}$$

$$\xi_{ke} \sim \text{Normal}(0, \rho_{\lambda e}^2)$$

3.2 Dimensionality adaptation

To adapt the structural complexity of the dynamical model for prototype signatures, we introduce dimensionality changing variables, $c_{k\mu}$ and $c_{k\lambda}$. These variables are both discrete-valued, and act as "switches" to control the number of distinct equilibrium levels or relaxation time parameters utilized by each prototype signature.

The variable $c_{k\mu}$ specifies one of four configurations for the equilibrium levels:

$$c_{k\mu} = 1 \Rightarrow \delta_{k\mu_{a\to c}} = \delta_{k\mu_{c\to e}} = 0 \Rightarrow \mu_{ka} = \mu_{kc} = \mu_{ke}$$

$$c_{k\mu} = 2 \Rightarrow \delta_{k\mu_{a\to c}} \neq 0, \delta_{k\mu_{c\to e}} = 0 \Rightarrow \mu_{ka} \neq \mu_{kc} = \mu_{ke}$$

$$c_{k\mu} = 3 \Rightarrow \delta_{k\mu_{a\to c}} = 0, \delta_{k\mu_{c\to e}} \neq 0 \Rightarrow \mu_{ka} = \mu_{kc} \neq \mu_{ke}$$

$$c_{k\mu} = 4 \Rightarrow \delta_{k\mu_{a\to c}} \neq 0, \delta_{k\mu_{c\to e}} \neq 0 \Rightarrow \mu_{ka} \neq \mu_{kc} \neq \mu_{ke}$$

The variable $c_{k\lambda}$ specifies one of two configurations for the relaxation time parameters:

$$c_{k\lambda} = 1 \Rightarrow \xi_{ke} = 0 \Rightarrow \lambda_{kc} = \lambda_{ke}$$

$$c_{k\lambda} = 2 \Rightarrow \xi_{ke} \neq 0 \Rightarrow \lambda_{kc} \neq \lambda_{ke}$$

4 Priors

4.1 Specification

For the parameters ϵ_1 and ϵ_2, which control the NBD noise model variance, we assume a normal prior on transformed values of ϵ_1 and ϵ_2, with hyperparameters m_ϵ and σ_ϵ:

$$\epsilon_1 = e^{\xi_{\epsilon_1}} \quad \text{s.t.} \quad \xi_{\epsilon_1} \sim \text{Normal}(m_{\epsilon_1}, \sigma^2_{\epsilon_1})$$

$$\epsilon_2 = e^{\xi_{\epsilon_2}} \quad \text{s.t.} \quad \xi_{\epsilon_2} \sim \text{Normal}(m_{\epsilon_2}, \sigma^2_{\epsilon_2})$$

For α, the DP concentration parameter, we assume a gamma prior with hyperparameters $\boldsymbol{\omega_\alpha}$:

$$\alpha \sim \text{Gamma}(\boldsymbol{\omega_\alpha})$$

For β_0, the mean for pre-antibiotic equilibrium levels μ_{k0}, we assume a normal prior with hyperparameters m_{β_0} and σ_{β_0}:

$$\beta_0 \sim \text{Normal}(m_{\beta_0}, \sigma^2_{\beta_0})$$

For ρ_0, the variance for pre-antibiotic equilibrium levels μ_{k0}, we assume an inverse χ^2 distribution with hyperparameters υ_{ρ_0} and ν_{ρ_0}:

$$\rho_0 \sim \text{Inv-}\chi^2(\upsilon_{\rho_0}, \nu_{\rho_0})$$

For ρ_μ and ρ_X, the variances for $\delta_{k\mu}$ and δ_{kX} variables, we assume inverse χ^2 distributions with corresponding υ and ν hyperparameters:

$$\rho_\mu \sim \text{Inv-}\chi^2(\upsilon_{\rho_\mu}, \nu_{\rho_\mu})$$

$$\rho_X \sim \text{Inv-}\chi^2(\upsilon_{\rho_X}, \nu_{\rho_X})$$

For β_{λ_c}, the mean for ξ_{kc}, which controls the relaxation time on interval (c), we assume a normal distribution with hyperparameters $m_{\beta_{\lambda_c}}$ and $\sigma_{\beta_{\lambda_c}}$:

$$\beta_{\lambda_c} \sim \text{Normal}(m_{\beta_{\lambda_c}}, \sigma^2_{\beta_{\lambda_c}})$$

For ρ_{λ_c}, the variance for ξ_{kc}, we assume an inverse χ^2 distribution with hyperparameters $v_{\rho_{\lambda_c}}$ and $\nu_{\rho_{\lambda_c}}$:

$$\rho_{\lambda_c} \sim \text{Inv-}\chi^2(v_{\rho_{\lambda_c}}, \nu_{\rho_{\lambda_c}})$$

For ρ_{λ_e}, the variance for ξ_{ke}, we assume an inverse χ^2 distribution with hyperparameters $v_{\rho_{\lambda_e}}$ and $\nu_{\rho_{\lambda_e}}$:

$$\rho_{\lambda_e} \sim \text{Inv-}\chi^2(v_{\rho_{\lambda_e}}, \nu_{\rho_{\lambda_e}})$$

For $c_{k\mu}$ and $c_{k\lambda}$, the parameters controlling dimensionality of prototype signature dynamics models, we assume a multinomial distribution with parameters $\boldsymbol{\pi}_\mu$ and $\boldsymbol{\pi}_\mu$, and Dirichlet distribution priors with hyperparameters $\boldsymbol{\omega}_{\pi_\mu}$ and $\boldsymbol{\omega}_{\pi_\lambda}$.

4.2 Setting hyperparameters

For the concentration parameter $\alpha \sim \text{Gamma}(\boldsymbol{\omega_\alpha})$, we set its hyperparameters $\omega_{\alpha 1} = \omega_{\alpha 2} = 10^{-5}$ for a weak prior with mean 1 and variance 10^5. See Section 7.1 for a sensitivity analysis of these hyperparameters.

We set the means and variances of prior distributions empirically from the data as follows. To set the hyperparameters, m_ϵ and σ_ϵ, specifying the priors for the variables controlling the NBD variances in the data noise model, we first computed maximum likelihood estimates for NBD variance parameters using the antibiotic exposure time-points for each refOTU. We then log transformed these estimates, and set $m_{\epsilon 1}$ and $\sigma_{\epsilon 1}$ equal to the means and variances of the log transformed estimates. The same procedure was done, using the pre-antibiotic exposure time-points, to set $m_{\epsilon 2}$ and $\sigma_{\epsilon 2}$.

We set the hyperparameters specifying priors for the variables controlling the prototype signature dynamical models as follows. We first log transformed the counts data after adding a small increment of $\iota = 0.25$ to avoid taking the log of zero.

For the antibiotic exposure interval (a), we computed the mean, $\widehat{\mu}_{soa}$ and variance $\widehat{\sigma}^2_{soa}$ of the transformed data for each refOTU o in each subject s. We set $m_{\beta_0} = \text{mean}(\widehat{\mu}_{soa})$ and $\sigma^2_{\beta_0} = \text{var}(\widehat{\mu}_{soa})$.

For the two post-antibiotic exposure intervals (c) and (e), we performed a non-linear least squares fit to estimate the parameters in equation 2 or 3 on the transformed data for each refOTU in each subject. The estimates for the equilibrium levels $\widehat{\mu}_{soc}$ and $\widehat{\mu}_{soe}$, and relaxation time parameters $\widehat{\lambda}_{soc}$ and $\widehat{\lambda}_{soe}$ were then used to set σ_{ρ_μ}, $m_{\beta_{\lambda_c}}$, $\sigma_{\beta_{\lambda_c}}$, and $\sigma_{\beta_{\lambda_e}}$ respectively, using the means or variances of the estimated parameters.

For the antibiotic exposure intervals (b) and (d), we computed the means, $\widehat{X}_{sob}$ and $\widehat{X}_{sod}$, for each refOTU and subject. We then set $\sigma^2_{\rho X}$ equal to the variance of estimated increments $\widehat{X}_{sob} - \widehat{\mu}_{soa}$ and $\widehat{X}_{sod} - \widehat{\mu}_{soc}$.

The hyperparameter ν in the inverse χ^2 distribution can be interpreted as a prior sample size. We set $\nu = 5$, a small prior sample size, for a weak prior.

The hyperparameters $\boldsymbol{\omega}_{\pi_\mu}$ and $\boldsymbol{\omega}_{\pi_\lambda}$ control the Dirichlet prior on the dimensionality changing variables $c_{k\mu}$ and $c_{k\lambda}$. These hyperparameters can be interpreted as prior sample sizes. We set $\boldsymbol{\omega}_{\pi_\mu} = (1, 1, 1, 1)$ and $\boldsymbol{\omega}_{\pi_\lambda} = (1, 1)$, to yield symmetric Dirichlet distributions specifying a prior sample size of 1. See Section 7.1 for a sensitivity analysis of these hyperparameters.

5 Model inference

The inference task for MC-TIMME is to estimate the posterior distribution of all model variables given the observed data. We developed an efficient Markov Chain Monte Carlo (MCMC) algorithm for this task. Our solution uses a combination of Gibbs sampling, Metropolis-Hastings (MH)/Reversible Jump (RJ), and auxiliary variable augmentation steps.

The MCMC sampling loop is as follows:

1. Sample z_{so}, the assignment of refOTU o in subject s to a prototype signature.
2. Sample prototype signature dynamical model variables μ_{k0}, $\boldsymbol{\delta}_k$, $\boldsymbol{\lambda}_k$, $c_{k\mu}$ and $c_{\lambda\mu}$.
3. Sample variables ϵ_1 and ϵ_2 controlling NBD variance.
4. Sample α, the DP concentration parameter.
5. Sample $\boldsymbol{\beta}$ and $\boldsymbol{\rho}$ variables controlling means and variances of dynamical model variables.
6. Sample $\boldsymbol{\pi}_\mu$ and $\boldsymbol{\pi}_\mu$ variables specifying the distribution on dimensionality changing variables.

5.1 Step 1: sampling assignments of refOTUs to prototype signatures

For the first step in the MCMC loop, we use the Gibbs sampling method outlined in [2]. We sample the assignment z_{so} for refOTU o in subject s, conditional on all the data and other model variables including assignments of the other refOTUs. In this step there are two cases: refOTU o in subject s is assigned to an existing prototype signature k, or it is assigned to a new prototype signature. The update equations are then:

$$\mathrm{p}(\mathsf{z}_{so} = k \mid \mathbf{z}_{-so}, \alpha, \boldsymbol{\Theta}, \mathbf{y}) \propto \frac{n_k^{-so}}{O-1+\alpha}\mathrm{p}(\mathbf{y}_{so} \mid \boldsymbol{\theta}_k) \quad \text{for } n_k^{-so} > 0 \tag{4}$$

$$\mathrm{p}(\mathsf{z}_{so} \neq \mathsf{z}_l, \forall\, l \neq so \mid \mathbf{z}_{-so}, \alpha, \boldsymbol{\Theta}, \mathbf{y}) \propto \frac{\alpha}{O-1+\alpha}\mathrm{p}(\mathbf{y}_{so} \mid \boldsymbol{\theta}_*) \tag{5}$$

Here, n_k^{-so} is the number of refOTUs assigned to prototype signature k excluding refOTU o in subject s, $\mathbf{z}_{-so}$ is the assignments of all refOTUs excluding refOTU o in subject s, and O is the total number of refOTUs across all subjects. The variable $\boldsymbol{\theta}_*$ represents the parameters for a new prototype signature. The data likelihood for $\mathbf{y}_{so}$ is given by:

$$\mathrm{p}(\mathbf{y}_{so} \mid \boldsymbol{\theta}_k) = \prod_{t=1}^{T_s} \text{Neg-Bin}(y_{sot};\ e^{f(t,\boldsymbol{\theta}_k)+\gamma_{so}+\psi_{st}}, \boldsymbol{\epsilon})$$

The Gibbs sampling procedure thus either assigns the refOTU to an existing prototype signature, or generates a new prototype signature. Because the prior distribution for the prototype signature variables is non-conjugate, it is not possible to analytically integrate out $\boldsymbol{\theta}_*$. Thus, we follow the method outlined by [2], and sample $\boldsymbol{\theta}_*$ from its prior distribution. Because the sample from the prior distribution is unbiased, this method will sample correctly from the underlying posterior distribution.

5.2 Step 2: sampling prototype signature shape variables

For the second step in the MCMC loop, sampling prototype signature shape variables, μ_{k0}, $\boldsymbol{\delta}_k$, $\boldsymbol{\lambda}_k$, $c_{k\mu}$ and $c_{k\lambda}$, we use MH/RJ steps.

For a particular prototype signature k, conditional on $\boldsymbol{\lambda}_k$ and $\boldsymbol{c}_k$, we have a Generalized Linear Model (GLM) using the NBD. We can write the parameterization of prototype signature k at time t in terms of canonical GLM parameters as:

$$h(e^{\boldsymbol{\eta}_k(t)}) = \boldsymbol{\eta}_k(t) = \boldsymbol{A}_{kt}(\boldsymbol{\lambda}_k, \boldsymbol{c}_k)' \boldsymbol{G}_{kt}(\mu_{k0}, \boldsymbol{\delta}_k, \boldsymbol{c}_k)$$

The NBD variance is given by:

$$b(e^{\boldsymbol{\eta}_k(t)}) = e^{\boldsymbol{\eta}_k(t)} + e^{2\boldsymbol{\eta}_k(t)} \epsilon_i$$

Here, $h(\cdot)$ denotes the link function, which in this case equals the natural logarithm function. The variable $\boldsymbol{A}_{kt}$ is a vector of regression coefficients that depends on the relaxation time and dimensionality changing parameters, and $\boldsymbol{G}_{kt}$ is the vector of independent variables for the GLM. Note that $\boldsymbol{A}_{kt}$ and $\boldsymbol{G}_{kt}$ will change dimension depending on the setting of $\boldsymbol{c}_k$.

We leverage this formulation of the model as a conditional GLM to generate good linear approximations to use as MH proposal distributions, using the method described by [3]. These approximations are directly connected to Weighted Least Squares (WLS) methods, which are often used for maximum likelihood estimates for GLM parameters.

The procedure for creating a WLS estimate is as follows. We first create transformed data values $\widetilde{\mathsf{y}}_{sot}$ for all data points assigned to prototype signature k:

$$\widetilde{\mathsf{y}}_{sot} = \boldsymbol{\eta}_k(t) + (\mathsf{y}_{sot} - e^{\boldsymbol{\eta}_k(t)+\gamma_{so}+\psi_{st}}) h'(e^{\boldsymbol{\eta}_k(t)+\gamma_{so}+\psi_{st}}) \tag{6}$$

We then use the transformed data values to generate a diagonal weight matrix $\boldsymbol{W}_k$:

$$W_{ksot}^{-1} = b(e^{\boldsymbol{\eta}_k(t)+\gamma_{so}+\psi_{st}}) \{h'(e^{\boldsymbol{\eta}_k(t)+\gamma_{so}+\psi_{st}})\}^2 \tag{7}$$

The moments for the proposal distribution used in the MH steps are then given by:

$$\begin{aligned} \boldsymbol{m}_k^{(j-1\to j)} &= \{(\boldsymbol{\Lambda}^{-1})^{(j-1\to j)} + \boldsymbol{A}_k'^{(j-1\to j)} \boldsymbol{W}_k^{(j-1\to j)} \boldsymbol{G}_k^{(j-1\to j)} \boldsymbol{A}_k^{(j-1\to j)}\} \times \\ &\quad \{(\boldsymbol{\Lambda}^{-1})^{(j-1\to j)} \boldsymbol{d}^{(j-1\to j)} + \boldsymbol{A}_k'^{(j-1\to j)} \boldsymbol{W}_k^{(j-1\to j)} \widetilde{\mathbf{y}}^{(j-1\to j)}\} \end{aligned} \tag{8}$$

$$\boldsymbol{C}_k^{(j-1\to j)} = \{(\boldsymbol{\Lambda}^{-1})^{(j-1\to j)} + \boldsymbol{A}_k'^{(j-1\to j)} \boldsymbol{W}_k^{(j-1\to j)}\}^{-1} \tag{9}$$

The vector $\boldsymbol{d}$ represents the prior mean for the GLM, i.e., $d_1 = \beta_0$ and zero otherwise. The matrix $\boldsymbol{\Lambda}$ is the prior covariance for the GLM, i.e., $\Lambda_{11} = \rho_0^2$ and zero otherwise.

The MCMC iteration number is indexed by j, and the notation $(j-1 \to j)$ indicates a move from state $j-1$ to j in the MCMC sampler. To match dimensionality, the dimension of the target state is always used, e.g., $\boldsymbol{A}_k^{(j-1\to j)} = \boldsymbol{A}(\boldsymbol{\lambda}_k^{(j-1)}, \boldsymbol{c}_k^{(j)})$.

For each prototype signature k, the MH/RJ move from state $j-1$ to j will then sample $\boldsymbol{c}_k^{(j)}$, $\boldsymbol{\lambda}_k^{(j)}$, $\mu_{k0}^{(j)}$, and $\boldsymbol{\delta}_k^{(j)}$ as follows:

1. Sample $c_{k\mu}^{(j)}$ and $c_{k\lambda}^{(j)}$ from symmetric multinomial proposal distributions.

2. Sample $\xi_{k\lambda_c}$ from a normal proposal distribution with mean $\xi_{k\lambda_c}^{(j-1)}$ and fixed variance $\sigma^2_{\lambda_c}\kappa_{\lambda_c}$. If $c_{k\lambda} = 2$, then sample ξ_{λ_e} from a normal proposal distribution with mean $\xi_{k\lambda_e}^{(j-1)}$ and fixed variance $\sigma^2_{\lambda_e}\kappa_{\lambda_e}$. Otherwise, set $\lambda_{ke} = 0$.

3. Calculate the WLS proposal distribution moments $\boldsymbol{m}_k^{(j-1\to j)}$ and $\boldsymbol{C}_k^{(j-1\to j)}$ as described above, and sample $\mu_{k0}^{(j)}$ and $\boldsymbol{\delta}_k^{(j)}$ from the proposal distribution Normal$(\boldsymbol{m}_k^{(j-1\to j)}, \boldsymbol{C}_k^{(j-1\to j)})$.

Here, κ_λ are parameters used to tune the acceptance rate to ≈ 0.23, the conventionally used acceptance rate for multi-dimensional MH (see [4] for details).

The acceptance probability $q(j-1 \to j)$ for the move is given by:

$$q(j-1 \to j) = \min\left\{1, \frac{q_1}{q_2}\right\}$$

$$q_1 = l_k(\mathbf{y};\ \mu_{k0}^{(j)}, \boldsymbol{\delta}_k^{(j)}, \boldsymbol{\lambda}_k^{(j)}, \boldsymbol{c}_k^{(j)}, \boldsymbol{\epsilon}) \cdot \mathrm{p}(\mu_{k0}^{(j)}, \boldsymbol{\delta}_k^{(j)}, \boldsymbol{\lambda}_k^{(j)}, c_{k\mu}^{(j)}, c_{k\lambda}^{(j)} \mid \boldsymbol{\beta}, \boldsymbol{\rho}, \boldsymbol{\pi}_\mu, \boldsymbol{\pi}_\lambda) \cdot \mathcal{J}^{(j-1\leftarrow j)}$$

$$q_2 = l_k(\mathbf{y};\ \mu_{k0}^{(j-1)}, \boldsymbol{\delta}_k^{(j-1)}, \boldsymbol{\lambda}_k^{(j-1)}, \boldsymbol{c}_k^{(j-1)}, \boldsymbol{\epsilon}) \cdot \mathrm{p}(\mu_{k0}^{(j-1)}, \boldsymbol{\delta}_k^{(j-1)}, \boldsymbol{\lambda}_k^{(j-1)}, c_{k\mu}^{(j-1)}, c_{k\lambda}^{(j-1)} \mid \boldsymbol{\beta}, \boldsymbol{\rho}, \boldsymbol{\pi}_\mu, \boldsymbol{\pi}_\lambda) \cdot \mathcal{J}^{(j-1\to j)}$$

Here, $\mathrm{p}(\mu_{k0}^{(j)}, \boldsymbol{\delta}_k^{(j)}, \boldsymbol{\lambda}_k^{(j)}, c_{k\mu}^{(j)}, c_{k\lambda}^{(j)} \mid \boldsymbol{\beta}, \boldsymbol{\rho}, \boldsymbol{\pi}_\mu, \boldsymbol{\pi}_\lambda)$ is the product of prior distributions for μ_k, $\boldsymbol{\delta}_k$, $\boldsymbol{\lambda}_k$, $c_{k\mu}$ and $c_{k\lambda}$, as described in Section 3.

The term $\mathcal{J}^{(j-1\to j)}$ indicates the jumping probability, and is given by the product of the proposal distributions for $\xi_{k\boldsymbol{\lambda}}^{(j)}$, $\mu_{k0}^{(j)}$ and $\boldsymbol{\delta}_k^{(j)}$ starting from $\xi_{k\boldsymbol{\lambda}}^{(j-1)}$, $\mu_{k0}^{(j-1)}$ and $\boldsymbol{\delta}_k^{(j-1)}$. The reverse jump, $\mathcal{J}^{(j-1\leftarrow j)}$ is calculated analogously, by forming the product of the proposal distributions for $\xi_{k\boldsymbol{\lambda}}^{(j-1)}$, $\mu_{k0}^{(j-1)}$ and $\boldsymbol{\delta}_k^{(j-1)}$ starting from $\xi_{k\boldsymbol{\lambda}}^{(j)}$, $\mu_{k0}^{(j)}$ and $\boldsymbol{\delta}_k^{(j)}$.

The function $l_k(\mathbf{y};\ \mu_{k0}\boldsymbol{\delta}_k, \boldsymbol{\epsilon})$ is the data likelihood restricted to data assigned to prototype signature k:

$$l_k(\mathbf{y};\ \mu_{k0}, \boldsymbol{\delta}_k, \boldsymbol{\epsilon}) = \prod_{s,o\,:\,z_{so}=k} \prod_t \text{Neg-Bin}(\mathsf{y}_{so};\ e^{\eta_k(t)+\gamma_{so}+\psi_{st}}, \boldsymbol{\epsilon})$$

5.3 Step 3: sampling variables controlling NBD variance

We use MH steps for sampling ϵ_1 and ϵ_2. In this case, because we are sampling a single variable, we use a simple proposal distribution of Normal$(\xi_{\epsilon_i}^{(j-1)}, \kappa_{\epsilon_i}\sigma^2_{\epsilon_i})$ such that $\epsilon_i^{(j-1)} = e^{\xi_{\epsilon_i}^{(j-1)}}$. The parameter κ_{ϵ_i} is used to tune the acceptance rate to ≈ 0.44, the conventionally used acceptance rate for single dimensional MH (see [4] for details).

The acceptance probability for the ϵ_i MH steps is given by:

$$q(\epsilon_i^{(j-1)}, \epsilon_i^{(j)}) = \min\left\{1, \frac{l(\mathbf{y};\ \boldsymbol{\Theta}^{(j)}) \cdot \text{Normal}(\xi_{\epsilon_i}^{(j)};\ m_{\epsilon_i}, \sigma_{\epsilon_i}) \cdot \text{Normal}(\xi_{\epsilon i}^{(j-1)};\ \xi_{\epsilon_i}^{(j)}, \kappa_{\epsilon_i}\sigma^2_{\epsilon_i})}{l(\mathbf{y};\ \boldsymbol{\Theta}^{(j-1)}) \cdot \text{Normal}(\xi_{\epsilon_i}^{(j-1)};\ m_{\epsilon_i}, \sigma_{\epsilon_i}) \cdot \text{Normal}(\xi_{\epsilon i}^{(j)};\ \xi_{\epsilon_i}^{(j-1)}, \kappa_{\epsilon_i}\sigma^2_{\epsilon_i})}\right\}$$

5.4 Step 4: sampling the concentration parameter

For sampling the concentration parameter α, we use an efficient auxiliary variable sampling method as described in [5].

5.5 Step 5: sampling means and variances of dynamics variables

Sampling $\boldsymbol{\beta}$ and $\boldsymbol{\rho}$ is accomplished via straightforward Gibbs sampling steps [4], as these variables have conjugate priors.

5.6 Step 6: sampling variables specifying the distribution on dimensionality changing variables

Sampling $\boldsymbol{\pi}_\mu$ and $\boldsymbol{\pi}_\mu$ is accomplished via straightforward Gibbs sampling steps [4], as these variables have conjugate priors.

6 Experimental design algorithm

We use a greedy optimization algorithm with the Bayesian *D*-optimality function $g(\cdot)$ defined in the main manuscript, to generate experimental designs (Figure 2).

```
GreedyExpDesign(T_0,T_c,Y,N,J)
    // T_0 is an initial set of time-points
    // T_c is the set of all time-points available
    // Y is a set of prior observations
    // N is the number of desired time-points in the design
    // J is the number of MCMC samples to use

    approximate p(Θ) with J MCMC samples using Y as data
    T ← T_0
    while |T| ≤ N
        find t_m ∈ T_c that maximizes g({T ∪ t_m}, Θ^(1), ..., Θ^(J))
        T_c ← {T_c \ t_m}
        T ← T ∪ t_m
    end
    return T
```

Figure 2: Pseudo-code for greedy experimental design algorithm.

Learning of sequential or cross-subject experimental designs was performed as follows. For sequential designs, a small set of initial time-points T_e was used for estimating hyper-parameters: the first and last days of each antibiotic treatment, the first and last days of the entire series, and the first day before and after each antibiotic treatment. MCMC samples were collected using the time-points T_e, which were then used as input into `GreedyExpDesign` to predict the next set of n time-points to sample. These time-points were then concatenated with T_e, and the process described above was repeated until the desired total number of time-points was selected. For efficiency, we chose $n = 6$-8, depending on the number of time-points sampled in the original dataset. For cross-subject designs, data from all time-points from subject A were used as input to MC-TIMME, and MCMC samples were collected. These MCMC samples were then used as input to `GreedyExpDesign`, to predict time-points to sample for subject B. This procedure was repeated for

all pairs of subjects A and B. For each strategy, we produced a design that used 75% of the time-points from the original experiments. To eliminate bias due to the chosen starting set T_e, we required all designs to include this set of time-points. To estimate predictive accuracy, we used values for all refOTUs at the selected time-points, to fit the MC-TIMME model, and then calculated predictive error on the remaining 25% of held-out time-points. We used root mean squared error (RMSE) as the measure of predictive error, which is the square root of the sum of squared differences between actual and predicted sequencing counts, averaged over the number of refOTUs and time-points.

7 Tests

7.1 Sensitivity testing of model dimensionality adaptation

The MC-TIMME model adapts in dimensionality in two ways: (1) the number of equilibrium levels and relaxation time parameters used by a prototype signature, and (2) the number of prototype signatures used to model the ecosystem. The first type of adaptation is controlled by the discrete-valued variables $c_{k\mu}$ and $c_{k\lambda}$ for each prototype signature k. These variables are multinomial distributed with parameter vectors $\boldsymbol{\pi}_\mu$ and $\boldsymbol{\pi}_\lambda$. We placed Dirichlet priors on the multinomial distributions. The second type of adaptation is governed by a Dirichlet Process with concentration parameter α. We placed a gamma prior with hyperparameters $\boldsymbol{\omega_\alpha}$ on the concentration parameter α. The expected number of prototype signatures K is given by $K \approx \alpha \ln(N/\alpha + 1)$ [7], where N is the total number of refOTU time-series (756 for the dataset we analyzed).

To test the sensitivity of MC-TIMME's dimensionality adaptation to hyperparameter settings, we varied the hyperparameters on $c_{k\mu}$, $c_{k\lambda}$, and α. For each test, we ran the algorithm 8 times, and then computed the median and 95% credible intervals for Signature Diversity scores. In addition, we computed Normalized Mutual Information (NMI) and Rand Index (RI) scores. The NMI and RI scores are external criteria of clustering quality, which measure how well a particular cluster (i.e., assignment of refOTU time-series to prototype signatures) compare to a gold standard [6]; NMI and RI values of 1 indicate perfect correspondance between the clusterings. In this case, we used 8 runs of MC-TIMME using the default hyperparameter settings as the gold standard. The median and credible intervals for the NMI and RI scores were computed based on all pairs of scores between the default runs and a given test run. Figure 3 shows the results of our sensitivity testing.

For $c_{k\mu}$, we tested a non-symmetric Dirichlet prior "skewed" toward $\pi_{\mu 1} = 0.75$ with the Dirichlet prior equal to $(3, 1, 1, 1)$, e.g., favoring no change in equilibrium levels, with $3 : 1$ odds instead of the default of $1 : 1$. Most of the signature diversity and NMI/RI score medians were virtually identical, and there were no significant deviations in scores as assessed by overlap of credible intervals. Only the SD1$_\lambda$ score showed a non-significant trend of being slightly elevated under the "skewed" prior.

For $c_{k\lambda}$, we tested a non-symmetric Dirichlet prior "skewed" toward $\pi_{\lambda 1} = 0.75$ with the Dirichlet prior equal to $(3, 1)$, e.g., favoring no change in the relaxation time parameter on the second antibiotic exposure interval, with $3 : 1$ odds instead of the default of $1 : 1$. All the signature diversity and NMI/RI score medians were virtually identical, and there were no significant deviations in scores as assessed by overlap of credible intervals.

For the concentration parameter α, we tested two extreme values for the gamma prior mean, 10^{-3} and 10^3. These settings represent 1000X decreases or increases over the default mean of 1. For both the 10^{-3} and 10^3 settings, most signature diversity and NMI/RI score medians were virtually identical, and there were

	NMI	RI	SD3	$SD1_\mu$			$SD1_\lambda$			SD2		
				D	E	F	D	E	F	D	E	F
**default**	0.92-0.95	0.99-0.99	0.48-0.49	0.75-0.79	0.45-0.52	0.71-0.75	0.80-0.97	0.75-0.97	0.79-0.97	22.7-23.4	10.0-10.3	16.6-17.5
**c_μ non-unif.**	0.91-0.95	0.98-0.99	0.48-0.49	0.75-0.78	0.45-0.51	0.71-0.73	0.89-0.95	0.90-0.97	0.90-0.96	22.9-23.6	10.1-10.6	17.0-17.6
**c_λ non-unif.**	0.91-0.95	0.99-0.99	0.48-0.49	0.76-0.78	0.45-0.50	0.71-0.73	0.75-0.94	0.69-0.93	0.73-0.95	22.6-23.6	9.9-10.5	16.7-17.3
**alpha 10^{-3}**	0.91-0.95	0.99-0.99	0.48-0.49	0.76-0.78	0.47-0.50	0.71-0.73	0.80-0.97	0.80-0.99	0.86-0.98	22.5-23.3	9.9-10.3	16.6-17.3
**alpha 10^{3}**	0.91-0.95	0.99-0.99	0.48-0.49	0.76-0.79	0.46-0.51	0.71-0.74	0.85-0.96	0.87-0.97	0.88-0.97	22.7-23.7	9.9-10.5	17.0-17.6

Figure 3: **Sensitivity testing of MC-TIMME dimensionality adaptation to hyperparameter settings.** Each test consists of 8 MCMC runs. Default = default hyperparameter settings, c_μ non-unif. = setting of Dirichlet prior on π_μ to $(3, 1, 1, 1)$, c_λ non-unif. = setting of Dirichlet prior on π_λ to $(3, 1)$, alpha 10^{-3} and 10^3 = setting of gamma prior mean on α to 10^{-3} or 10^3. NMI = Normalized Mutual Information, RI = Rand Index, SD3 = Signature Diversity type 3 score (inter-ecosystem), $SD1_\mu$ and $SD1_\lambda$ = Signature Diversity type 1 scores (intra-signature diversity) for subjects D-F, SD2 = Signature Diversity type 2 scores (intra-ecosystem diversity) for subjects D-F.

no significant deviations in scores as assessed by overlap of credible intervals.

Our tests indicate that the model dimensionality (complexity) inferred by MC-TIMME showed little sensitivity to changes in hyperparameter settings. A contributing factor to this result is the hierarchical design of the MC-TIMME model. The model does not require direct setting of parameters for priors on the variables affecting dimensionality adaptation, but instead uses a second level of hyperparameters. In general, such hierarchical Bayesian models are less sensitive to prior distribution choices, as the parameters of prior distributions are not fixed, and thus can adapt based on the underlying data [4].

7.2 Data simulations

We tested the ability of MC-TIMME to recover a "gold standard" set of signatures, by simulating data with different amounts of added noise. Because a gold standard experimental dataset was not available, we used the recovered signatures as described in the main manuscript as the reference signatures; we believe this represents a more realistic scenario than a "toy" example with a small number of signatures. For each level of noise, 8 simulated datasets were created by generating counts data from the reference signatures using a negative binomial distribution (NBD) noise model. The NBD parameters controlling variance were set equal to their empirically determined values from the Dethlefsen $et\ al.$ data ($1.0X$ setting, or $\approx 60\%$ coefficient of variation for counts). We also tested a lower level of noise ($0.5X$ setting) and a higher level of noise ($1.5X$ setting). We computed the percent error for Signature Diversity scores and median relaxation time parameter estimates, with 8 runs of the original dataset used as the gold standard. Normalized Mutual Information (NMI) and Rand Index (RI) scores were also computed, as described in the previous section.

Figures 4 and 5 depict the results of our data simulations. The simulations using noise levels equal to those in the original dataset ("1.0X ϵ") are indicative of the performance of MC-TIMME in the presence of a realistic amount of noise. However, noise levels in the original data are likely to be higher than in more recent 16S phylotyping data sets, as Dethlefsen $et\ al.$ produced data using the now obsolete Roche 454 FLX chemistry and with a relatively small number of sequencing reads per sample. In any case, the "1.0X ϵ" simulations produced error rates for Signature Diversity scores of $<\approx 10\%$, and for median relaxation time parameters on the first post-antibiotic exposure interval of $\approx 25 - 30\%$. The error rates for the median

relaxation time parameters on the second post-antibiotic exposure interval were higher, at $\approx 30 - 45\%$. These higher error rates were not unexpected, as the second post-antibiotic exposure intervals were sampled at considerably fewer time-points than the first in the original experiments. For all simulations, NMI scores were reduced by $<\approx 20\%$, and RI scores were reduced by $< 3\%$. The NMI score is likely to be more representative of signature assignment consistency; the RI score has been shown to "saturate" with many clusters in data [6]. We note that the number of prototype signatures was systematically lower with increasing amounts of noise (not shown), suggesting that some fine details of signature shapes were lost as noise was added.

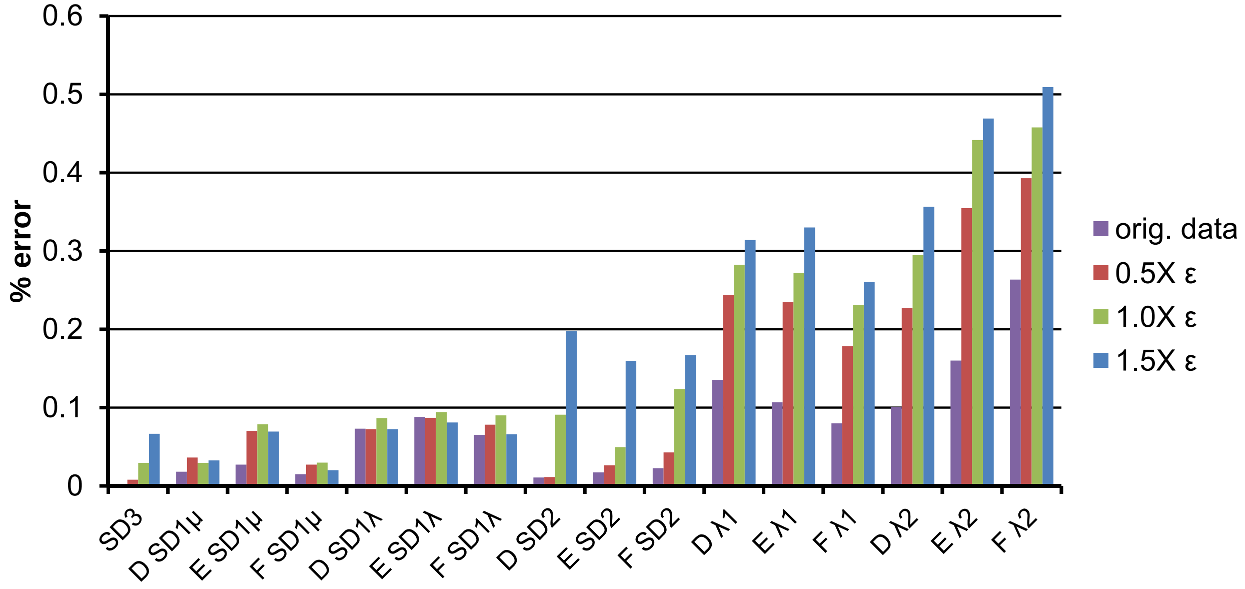

Figure 4: **Data simulations.** Each test consists of 8 MCMC runs. Orig. data = original data (not simulated), 0.5-1.5X ϵ = simulated data from reference signatures, with negative binomial distribution (NBD) parameters controlling variance set equal to 0.5, 1.0 or 1.5 times their empirically determined values from the Dethlefsen $et\ al.$ data. Each bar represents the percent error in estimating Signature Diversity scores or median relaxation time parameters, relative to the original dataset. SD3 = Signature Diversity type 3 score (inter-ecosystem), SD1_μ and SD1_λ = Signature Diversity type 1 scores (intra-signature diversity) for subjects D-F, SD2 = Signature Diversity type 2 scores (intra-ecosystem diversity) for subjects D-F, λ_1 = median relaxation time parameter for the first post-antibiotic exposure interval for subjects D-F, λ_2 = median relaxation time parameter for the second post-antibiotic exposure interval for subjects D-F.

Overall, our data simulations show that MC-TIMME can accurately estimate model parameters for complex microbial ecosystems in the presence of realistic amounts of experimental noise. Signature diversity scores exhibited the lowest error rates, likely because these scores are estimated using all refOTUs from each subject, or from all subjects in the case of the SD3 score. Relaxation time parameter estimates, which are estimated from prototype signatures comprised of potentially small groups of refOTUs, exhibited higher error rates. These results provide estimates of bounds on distinguishability of differences in model parameters in the presence of noise. However, we note that these results may vary with the complexity of the microbial ecosystems used as the reference "gold standard."

	NMI	% NMI med. reduction	RI	% RI med. reduction
**orig. data**	0.92-0.95	N/A	0.99-0.99	N/A
**0.5X ε**	0.81-0.84	11%	0.97-0.98	1%
**1.0X ε**	0.75-0.81	17%	0.97-0.98	2%
**1.5X ε**	0.71-0.76	22%	0.96-0.97	3%

Figure 5: **Data simulations.** Each test consists of 8 MCMC runs. Orig. data = original data (not simulated), 0.5-1.5X ϵ = simulated data from reference signatures, with negative binomial distribution (NBD) parameters controlling variance set equal to 0.5, 1.0 or 1.5 times their empirically determined values from the Dethlefsen $et\ al.$ data. NMI = Normalized Mutual Information, % NMI med. reduction = percent reduction in NMI relative to original data, RI = Rand Index, % RI med. reduction = percent reduction in RI relative to original data.

7.3 Subject effects

To test the effects on model estimation of excluding data from a particular subject, we ran MC-TIMME on all pairs of subjects. Each test consisted of 8 MCMC runs. We compared the test runs to 8 runs on the complete dataset, using all 3 subjects. Figures 6 and 7 display the results of these tests.

As depicted in Figure 6, the error rate for estimating Signature Diversity scores was $<\approx 10\%$ when subject D or F was excluded. However, the error rate was higher when subject E was excluded. The error rates for the relaxation time parameters followed similar trends, but were overall higher than for the Signature Diversity scores, as noted in the previous section. Subjects D and F were least similar, in terms of sharing of prototype signatures (SD3 score of 0.70), and subjects E and F were most similar (SD3 score of 0.31). Inclusion of subject E with subject D or F lowered the error rate, as subject E shared a substantial number of prototype signatures with both subjects D and F. This sharing of prototype signatures effectively increases statistical power, because estimates of model parameters are derived from groups of refOTUs rather than single instances.

Excluding any given subject did not affect the relative ordering of Signature Diversity scores. Further, the effect of excluding any subject was about the same as having a realistic level of noise present in the data, as shown in the previous section. Thus, given the level of noise in the dataset analyzed, MC-TIMME did not appear to lose resolution when data from one subject was excluded. However, studies with more subjects will be necessary to fully explore the effects of the number of subjects on model performance. For a large number of subjects, it may prove useful to extend MC-TIMME to use an adaptive Hierarchical Dirichlet Process prior to leverage similarities or differences among groups of subjects [8].

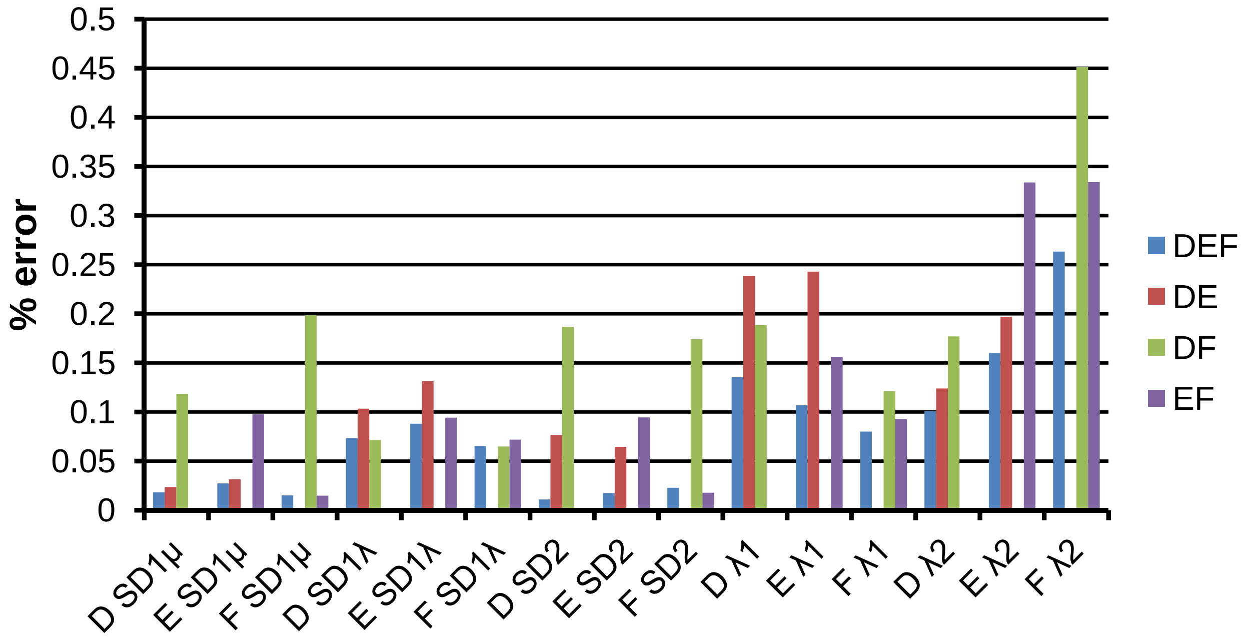

Figure 6: **Subjects effects.** Each test consists of 8 MCMC runs. Each label indicates the subjects that were included (e.g., DE = subjects D and E). Each bar represents the percent error in estimating Signature Diversity scores or median relaxation time parameters, relative to the original dataset in which all subjects were present (DEF). $SD1_\mu$ and $SD1_\lambda$ = Signature Diversity type 1 scores (intra-signature diversity) for subjects D-F, SD2 = Signature Diversity type 2 scores (intra-ecosystem diversity) for subjects D-F, λ_1 = median relaxation time parameter for the first post-antibiotic exposure interval for subjects D-F, λ_2 = median relaxation time parameter for the second post-antibiotic exposure interval for subjects D-F.

	NMI	RI	SD3	$SD1_\mu$			$SD1_\lambda$			SD2		
				D	E	F	D	E	F	D	E	F
**DEF**	0.92-0.95	0.99-0.99	0.48-0.49	0.75-0.79	0.45-0.52	0.71-0.75	0.80-0.97	0.75-0.97	0.79-0.97	22.7-23.4	10.0-10.3	16.6-17.5
**DE**	0.83-0.87	0.96-0.97	0.58-0.59	0.74-0.77	0.46-0.50	N/A	0.70-0.94	0.65-0.92	N/A	20.8-21.8	9.2-9.7	N/A
**DF**	0.78-0.82	0.98-0.98	0.69-0.70	0.84-0.89	N/A	0.82-0.90	0.69-0.96	N/A	0.68-0.97	18.4-19.0	N/A	13.9-14.4
**EF**	0.77-0.83	0.98-0.99	0.30-0.32	N/A	0.41-0.46	0.71-0.73	N/A	0.56-0.98	0.63-0.98	N/A	9.0-9.3	16.8-17.2

Figure 7: **Subject effects.** Each test consists of 8 MCMC runs. Row labels indicate the subjects that were included (e.g., DE = subjects D and E). NMI = Normalized Mutual Information, RI = Rand Index, SD3 = Signature Diversity type 3 score (inter-ecosystem; note that this applies only to the included subjects), $SD1_\mu$ and $SD1_\lambda$ = Signature Diversity type 1 scores (intra-signature diversity) for subjects D-F, SD2 = Signature Diversity type 2 scores (intra-ecosystem diversity) for subjects D-F.

References

[1] Sethuraman, J. A Constructive Definition of Dirichlet Priors. *Statistica Sinica* **4**, 639–650 (1994).

[2] Rasmussen, C. The Infinite Gaussian Mixture Model. In *Neural Information Processing Systems (NIPS)* (2000).

[3] Gamerman, D. Sampling from the posterior distribution in generalized linear mixed models. *Statistics and Computing* **7**, 57–68 (1997).

[4] Gelman, A., Carlin, J., Stern, H. & Rubin, D. *Bayesian Data Analysis* (Chapman & Hall/CRC, 2004).

[5] Escobar, M. D. & West, M. Bayesian Density Estimation and Inference Using Mixtures. *Journal of the American Statistical Association* **90**, 577–588 (1995).

[6] Antoniak, C. Mixtures of Dirichlet processes with applications to Bayesian nonparametric problems. *Annals of Statistics* **2**, 1152–1174 (1974).

[7] Manning, C., Prabhakar, R. & Schutze, H. *Introduction to Information Retrieval* (Cambridge University Press, 2004).

[8] Gerber, G. K., Dowell, R. D., Jaakkola, T. S. & Gifford, D. K. Automated discovery of functional generality of human gene expression programs. *PLoS Computational Biology* **3(8)**, e148 (2007).
